# Supplementary material for: The performance of partially substituted composite ester materials with weathered red-bed soil in ecological restoration
Source: PLoS One. 2024 Apr 3;19(4):e0299323. doi: 10.1371/journal.pone.0299323 (PMC10990215; doi:10.1371/journal.pone.0299323)
Supplement: S1 File — (DOCX) [file pone.0299323.s001.docx]

# Fig 1. (a)Relative mineral content

| **Quartz（%）** | **Illite (%)** | **Kaolinite (%)** | **Chlorite (%)** | **Montmorillonite (%)** | **Anorthite (%)** | **Hematite (%)** |
| --- | --- | --- | --- | --- | --- | --- |
| 66.32 | 10.47 | 13.36 | 4.27 | 0.38 | 0.50 | 4.69 |

# Fig 1. (b)Grain size distribution curve

| **Particle size（mm）** | >5.000 | 2.000-5.000 | 1.000-2.000 | 0.500-1.000 | 0.250-0.500 | 0.100-0.250 | 0.075-0.100 | <0.075 |
| --- | --- | --- | --- | --- | --- | --- | --- | --- |
| **Grain size distribution** | 16.000 | 26.300 | 51.500 | 112.900 | 105.500 | 54.000 | 100.200 | 34.000 |

# 25th day average plant height (mm)

| **Group number** | **Plant height on the 25th day (mm)** | | | |
| --- | --- | --- | --- | --- |
|  | **Measurement 1** | **Measurement 2** | **Measurement 3** | **Mean value** |
| 1 | 59 | 62 | 62 | 61.00 |
| 2 | 75 | 75 | 75 | 75.00 |
| 3 | 51 | 49 | 50 | 50.00 |
| 4 | 64 | 59 | 57 | 60.00 |
| 5 | 53 | 53 | 50 | 52.00 |
| 6 | 40 | 40 | 40 | 40.00 |
| 7 | 51 | 48 | 51 | 50.00 |
| 8 | 60 | 63 | 60 | 61.00 |
| 9 | 42 | 42 | 45 | 43.00 |
| 10 | 40 | 38 | 42 | 40.00 |

# Fig 5. (a) Plant growths.

| **Group number** | **Germination rate (%)** | **Plant height on the 25th day (mm)** |
| --- | --- | --- |
| 1 | 67.00 | 61.00 |
| 2 | 79.00 | 75.00 |
| 3 | 72.00 | 50.00 |
| 4 | 68.00 | 60.00 |
| 5 | 72.00 | 52.00 |
| 6 | 63.00 | 40.00 |
| 7 | 65.00 | 50.00 |
| 8 | 65.00 | 61.00 |
| 9 | 58.00 | 43.00 |
| 10 | 57.00 | 40.00 |

# Fig 5. (b) Soil parameters.

| **Soil parameters** | **Water-absorbing content / Adhesive content (g/m^2^)** | | | |
| --- | --- | --- | --- | --- |
|  | **0.00/0.00** | **15.00/5.00** | **30.00/5.00** | **60.00/10.00** |
| **Shrinkage rate (%)** | 31.03 | 31.29 | 30.23 | 28.48 |
| **Internal friction angle (°)** | 14.8 | 17.3 | 26.7 | 22.7 |
| **Cohesion (kPa)** | 11.04 | 27.02 | 17.91 | 10.72 |
| **Erosion rate (%)** | 3.47 | 14.44 | 16.35 | 17.3 |
| **Water content (%)** | 13.32 | 13.45 | 11.98 | 10.47 |
| **Swelling rate (%)** | 10.58 | 9.72 | 8.45 | 7.26 |
| **pH** | 5.6 | 6.5 | 6.8 | 6.8 |
| **Permeability coefficient (cm/s)** | 0.78 | 0.66 | 0.38 | 0.31 |
| **Electrical conductivity (mS/cm)** | 0.54 | 0.72 | 0.44 | 0.24 |

# Erosion rate calculation

| **Water-absorbing content:60.00 g/m2; Adhesive content:10.00 g/m2:** | | | | | |
| --- | --- | --- | --- | --- | --- |
| Weathered red-bed soil (%) | 0 | 1 | 2.5 | 5 | 10 |
| Gross weight (g) | 317.4 | 900.8 | 917.9 | 322.6 | 317.4 |
| Skin weight (g) | 278 | 822.1 | 869.1 | 277.5 | 278 |
| net weight (g) | 63.2 | 78.7 | 48.8 | 45.1 | 39.4 |
| Erosion rate (%) | 3.47 | 3.935 | 2.44 | 2.255 | 1.97 |
| **Water-absorbing content:30.00 g/m2; Adhesive content:5.00 g/m2:** | | | | | |
| Weathered red-bed soil (%) | 0 | 1 | 2.5 | 5 | 10 |
| Gross weight (g) | 1240.3 | 1062.6 | 1549.8 | 915.7 | 990.8 |
| Skin weight (g) | 951.6 | 870.1 | 1469.1 | 823 | 952.5 |
| net weight (g) | 288.7 | 192.5 | 80.7 | 92.7 | 38.3 |
| Erosion rate (%) | 14.435 | 9.625 | 4.035 | 4.635 | 1.915 |
| **Water-absorbing content:15.00 g/m2; Adhesive content:2.50 g/m2:** | | | | | |
| Weathered red-bed soil (%) | 0 | 1 | 2.5 | 5 | 10 |
| Gross weight (g) | 1688.4 | 1559.1 | 930.1 | 956.8 | 1020.2 |
| Skin weight (g) | 1467.8 | 1467.8 | 869.1 | 822.1 | 951.6 |
| net weight (g) | 220.6 | 91.3 | 61 | 134.7 | 68.6 |
| Erosion rate (%) | 11.03 | 4.565 | 3.05 | 6.735 | 3.43 |
| **Water-absorbing content:15.00 g/m2; Adhesive content:5.00 g/m2:** | | | | | |
| Weathered red-bed soil (%) | 0 | 1 | 2.5 | 5 | 10 |
| Gross weight (g) | 884.3 | 546.5 | 455.6 | 479.8 | 436.4 |
| Skin weight (g) | 557.4 | 281.5 | 277.5 | 277.1 | 278 |
| net weight (g) | 326.9 | 265 | 178.1 | 202.7 | 158.4 |
| Erosion rate (%) | 16.345 | 13.25 | 8.905 | 10.135 | 7.92 |
| **Water-absorbing content:30.00 g/m2; Adhesive content:2.50 g/m2:** | | | | | |
| Weathered red-bed soil (%) | 0 | 1 | 2.5 | 5 | 10 |
| Gross weight (g) | 841.6 | 1557.3 | 982.1 | 945.7 | 1028.5 |
| Skin weight (g) | 559 | 1467.8 | 869.1 | 822.1 | 951.6 |
| net weight (g) | 282.6 | 89.5 | 113 | 123.6 | 76.9 |
| Erosion rate (%) | 14.13 | 4.475 | 5.65 | 6.18 | 3.845 |

# Fig 6. Comparison of erosion rate (%)

| **materials** | **0.00/0.00 (g/m^2^)** | **15.00/2.50 (g/m^2^)** | **15.00/5.00 (g/m^2^)** | **30.00/2.50 (g/m^2^)** | **30.00/5.00 (g/m^2^)** | **60.00/10.00 (g/m^2^)** |
| --- | --- | --- | --- | --- | --- | --- |
| **0.00** **(%)** | 17.3 | 11.03 | 16.35 | 14.13 | 14.435 | 3.47 |
| **1.00 (%)** | 6.856 | 4.565 | 13.25 | 4.475 | 9.625 | 3.935 |
| **2.50 (%)** | 12.563 | 3.05 | 8.91 | 5.65 | 4.035 | 2.44 |
| **5.00 (%)** | 6.365 | 6.735 | 10.14 | 6.18 | 4.635 | 2.255 |
| **10.00 (%)** | 5.869 | 3.43 | 7.92 | 3.845 | 1.915 | 1.97 |

# Fig 7. Comparison of the cohesive forces of each sample (%)

| **materials** | **0.00/0.00 (g/m^2^)** | **15.00/2.50 (g/m^2^)** | **15.00/5.00 (g/m^2^)** | **30.00/2.50 (g/m^2^)** | **30.00/5.00 (g/m^2^)** | **60.00/10.00 (g/m^2^)** |
| --- | --- | --- | --- | --- | --- | --- |
| **0.00 (%)** | 8.38 | 18.75 | 17.07 | 16.62 | 27.78 | 11.04 |
| **1.00 (%)** | 10.72 | 24.03 | 17.91 | 19.57 | 26.15 | 21.80 |
| **2.50 (%)** | 17.16 | 26.73 | 19.56 | 24.03 | 27.02 | 22.00 |
| **5.00 (%)** | 18.48 | 27.19 | 23.46 | 24.96 | 32.98 | 23.51 |
| **10.00 (%)** | 18.48 | 40.26 | 23.98 | 27.00 | 34.84 | 36.70 |

# Fig 8. Comparison of the internal friction angles of each sample (°)

| **materials** | **0.00/0.00 (g/m^2^)** | **15.00/2.50 (g/m^2^)** | **15.00/5.00 (g/m^2^)** | **30.00/2.50 (g/m^2^)** | **30.00/5.00 (g/m^2^)** | **60.00/10.00 (g/m^2^)** |
| --- | --- | --- | --- | --- | --- | --- |
| **0.00 (%)** | 22.70 | 20.30 | 26.70 | 23.90 | 17.30 | 14.80 |
| **1.00 (%)** | 20.10 | 14.70 | 14.70 | 16.40 | 14.30 | 24.40 |
| **2.50 (%)** | 6.10 | 8.20 | 10.50 | 15.40 | 22.80 | 20.60 |
| **5.00 (%)** | 14.90 | 16.60 | 20.60 | 13.70 | 25.10 | 28.10 |
| **10.00 (%)** | 15.60 | 17.70 | 23.40 | 27.50 | 18.60 | 16.60 |

# Fig 9. Line graphs showing the temporal change in moisture content of different samples.

## (1) Water-absorbing content:60.00 g/m^2^; Adhesive content:10.00 g/m^2^.

| Time(h) | Moisture content (%) | | | | |
| --- | --- | --- | --- | --- | --- |
|  | Weathered red-bed soil (%) of :0% | Weathered red-bed soil (%) of :1% | Weathered red-bed soil (%) of :2.5% | Weathered red-bed soil (%) of :5% | Weathered red-bed soil (%) of :10% |
| 0 | 50.81734 | 50.45423 | 49.78754 | 49.05792 | 50.31802 |
| 12 | -- | -- | -- | -- | -- |
| 24 | 39.23241 | 39.06359 | 40.43909 | 38.79972 | 40.28269 |
| 36 | -- | -- | -- | -- | -- |
| 48 | 31.8408 | 32.42488 | 34.49008 | 33.28681 | 33.85159 |
| 60 | 27.50533 | 29.21034 | 30.94901 | 30.63503 | 30.67138 |
| 72 | 23.52523 | 25.71628 | 27.54958 | 28.12282 | 27.70318 |
| 84 | 20.6823 | 23.20056 | 25.42493 | 26.37823 | 25.65371 |
| 96 | 17.19972 | 19.49686 | 22.23796 | 23.79623 | 22.89753 |
| 108 | 14.28571 | 16.28232 | 19.54674 | 21.28402 | 20.63604 |
| 120 | 11.08742 | 12.57862 | 16.07649 | 17.79484 | 17.73852 |
| 132 | 9.52381 | 10.48218 | 13.88102 | 15.49197 | 15.90106 |
| 144 | 7.9602 | 8.59539 | 10.90652 | 12.49128 | 13.35689 |
| 156 | 7.03625 | 7.54717 | 9.2068 | 10.39777 | 11.44876 |
| 168 | 6.25444 | 6.49895 | 7.79037 | 8.58339 | 9.46996 |
| 180 | 5.75693 | 5.87002 | 7.01133 | 7.53664 | 8.26855 |
| 192 | 5.18834 | 5.38085 | 6.30312 | 6.69923 | 7.42049 |
| 204 | 4.61976 | 4.8218 | 5.5949 | 5.93161 | 6.57244 |
| 216 | 4.12225 | 4.4724 | 5.24079 | 5.44313 | 5.9364 |
| 228 | 3.83795 | 4.05311 | 4.6034 | 4.88486 | 5.37102 |
| 240 | 3.62473 | 3.84347 | 4.39093 | 4.53594 | 4.947 |
| 252 | -- | -- | -- | -- | -- |
| 264 | 3.26937 | 3.3543 | 3.82436 | 3.76832 | 4.16961 |
| 276 | 3.12722 | 3.28442 | 3.6119 | 3.55897 | 3.88693 |
| 288 | 3.12722 | 3.28442 | 3.54108 | 3.48918 | 3.81625 |
| 300 | 2.98507 | 3.00489 | 3.32861 | 3.27983 | 3.53357 |
| 312 | 2.914 | 2.93501 | 3.11615 | 3.14027 | 3.25088 |
| 324 | 2.84293 | 2.79525 | 3.11615 | 3.0007 | 3.10954 |
| 336 | 2.84293 | 2.79525 | 3.04533 | 2.93091 | 3.03887 |
| 348 | 2.84293 | 2.72537 | 2.90368 | 2.86113 | 2.89753 |
| 360 | 2.84293 | 2.79525 | 2.9745 | 2.93091 | 2.89753 |
| 372 | 2.70078 | 2.79525 | 2.90368 | 2.86113 | 2.82686 |
| 384 | -- | -- | -- | -- | -- |
| 396 | 2.34542 | 2.30608 | 2.40793 | 2.30286 | 2.33216 |
| 408 | -- | -- | -- | -- | -- |
| 420 | 2.41649 | 2.37596 | 2.47875 | 2.37264 | 2.33216 |

## (2) Water-absorbing content:30.00 g/m2; Adhesive content:5.00 g/m2.

| Time(h) | Moisture content (%) | | | | |
| --- | --- | --- | --- | --- | --- |
|  | Weathered red-bed soil (%) of :0% | Weathered red-bed soil (%) of :1% | Weathered red-bed soil (%) of :2.5% | Weathered red-bed soil (%) of :5% | Weathered red-bed soil (%) of :10% |
| 0 | 50.43424 | 50.05938 | 49.90203 | 49.99626 | 50.17065 |
| 12 | -- | -- | -- | -- |  |
| 24 | 38.95198 | 39.86964 | 38.6897 | 39.56166 | 40.54608 |
| 36 | -- | -- | -- | -- |  |
| 48 | 32.34099 | 32.70169 | 32.80322 | 33.85342 | 34.19795 |
| 60 | 28.3048 | 28.55552 | 28.94898 | 30.25934 | 30.5802 |
| 72 | 24.26862 | 24.62017 | 25.37505 | 26.73573 | 27.09898 |
| 84 | 21.55463 | 21.87948 | 22.7822 | 24.41015 | 24.7099 |
| 96 | 18.07516 | 18.15495 | 19.34842 | 21.16843 | 21.56997 |
| 108 | 15.22199 | 14.99262 | 16.54534 | 18.27907 | 18.77133 |
| 120 | 11.95129 | 11.47892 | 13.18164 | 14.75546 | 15.22184 |
| 132 | 10.28114 | 9.65179 | 11.1494 | 12.50035 | 13.31058 |
| 144 | 8.68058 | 7.82467 | 9.11717 | 9.89288 | 10.64846 |
| 156 | 7.56715 | 6.77056 | 7.85578 | 8.27202 | 9.21502 |
| 168 | 6.73208 | 5.85699 | 7.01486 | 6.43975 | 7.52901 |
| 180 | 6.17537 | 5.15425 | 5.9637 | 6.01691 | 7.03072 |
| 192 | 5.68824 | 4.66233 | 5.40308 | 5.31219 | 6.41638 |
| 204 | 5.13152 | 4.02987 | 4.70231 | 4.60747 | 5.49147 |
| 216 | 4.85317 | 3.74877 | 4.35193 | 4.18464 | 5.25597 |
| 228 | 4.50522 | 3.32713 | 3.79131 | 3.7618 | 4.84642 |
| 240 | 4.29645 | 3.1163 | 3.72123 | 3.55039 | 4.7099 |
| 252 | -- | -- | -- | -- |  |
| 264 | 3.9485 | 2.69466 | 3.37085 | 3.19803 | 4.23208 |
| 276 | 3.80932 | 2.62439 | 3.30077 | 3.05708 | 4.09556 |
| 288 | 3.80932 | 2.55411 | 3.30077 | 2.98661 | 4.16382 |
| 300 | 3.67015 | 2.34329 | 3.16062 | 2.91614 | 3.95904 |
| 312 | 3.60056 | 2.34329 | 3.16062 | 2.91614 | 3.89078 |
| 324 | 3.53097 | 2.27301 | 3.09054 | 2.77519 | 3.82253 |
| 336 | 3.53097 | 2.27301 | 3.09054 | 2.77519 | 3.89078 |
| 348 | 3.46138 | 2.20274 | 3.02046 | 2.77472 | 3.75427 |
| 360 | 3.46138 | 2.27301 | 3.09054 | 2.77519 | 3.82253 |
| 372 | 3.46138 | 2.20274 | 3.09054 | 2.77519 | 3.82253 |
| 384 | -- | -- | -- | -- |  |
| 396 | 3.04384 | 1.7811 | 2.67008 | 2.75236 | 3.41297 |
| 408 | -- | -- | -- | -- |  |
| 420 | 3.04384 | 1.85137 | 2.6 | 2.72283 | 3.34471 |

## (3) Water-absorbing content:30.00 g/m2; Adhesive content:2.50 g/m2.

| Time(h) | Moisture content (%) | | | | |
| --- | --- | --- | --- | --- | --- |
|  | Weathered red-bed soil (%) of :0% | Weathered red-bed soil (%) of :1% | Weathered red-bed soil (%) of :2.5% | Weathered red-bed soil (%) of :5% | Weathered red-bed soil (%) of :10% |
| 0 | 50.55788 | 50.05783 | 50.39785 | 50.24289 | 50.70423 |
| 12 | -- | -- | -- | -- | -- |
| 24 | 40.86471 | 39.84485 | 41.72043 | 40.7356 | 41.05634 |
| 36 | -- | -- | -- | -- | -- |
| 48 | 33.4728 | 32.79267 | 35.41219 | 35.04511 | 35.70423 |
| 60 | 29.21897 | 28.98449 | 31.46953 | 31.5059 | 32.39437 |
| 72 | 25.31381 | 25.38787 | 27.8853 | 28.31367 | 29.22535 |
| 84 | 22.87308 | 22.84908 | 25.37634 | 26.02359 | 27.11268 |
| 96 | 19.45607 | 19.39351 | 22.07885 | 23.24774 | 24.29577 |
| 108 | 16.66667 | 16.57264 | 19.35484 | 20.81888 | 21.97183 |
| 120 | 13.38912 | 13.32863 | 15.98566 | 17.76544 | 18.94366 |
| 132 | 11.36681 | 11.14245 | 13.83513 | 15.75295 | 16.90141 |
| 144 | 9.13529 | 8.60367 | 11.03943 | 12.9771 | 14.08451 |
| 156 | 7.81032 | 7.19323 | 9.39068 | 10.89521 | 12.04225 |
| 168 | 6.7643 | 5.99436 | 7.8853 | 9.09091 | 9.85915 |
| 180 | 6.20642 | 5.21862 | 7.02509 | 7.98057 | 8.52113 |
| 192 | 5.64854 | 4.65444 | 6.16487 | 7.14781 | 7.32394 |
| 204 | 5.09066 | 4.16079 | 5.44803 | 6.45385 | 6.33803 |
| 216 | 4.60251 | 3.8787 | 4.94624 | 5.82929 | 5.70423 |
| 228 | 4.11437 | 3.52609 | 4.37276 | 5.13532 | 4.92958 |
| 240 | 3.9749 | 3.45557 | 4.08602 | 4.92713 | 4.50704 |
| 252 | -- | -- | -- | -- | -- |
| 264 | 3.48675 | 3.10296 | 3.51254 | 4.16378 | 3.73239 |
| 276 | 3.34728 | 3.03244 | 3.29749 | 3.88619 | 3.4507 |
| 288 | 3.34728 | 3.03244 | 3.29749 | 3.81679 | 3.4507 |
| 300 | 3.13808 | 2.8914 | 3.08244 | 3.40042 | 3.16901 |
| 312 | 2.99861 | 2.8914 | 3.01075 | 3.26162 | 3.09859 |
| 324 | 2.92887 | 2.82087 | 2.86738 | 3.12283 | 3.02817 |
| 336 | 2.85914 | 2.82087 | 2.86738 | 3.05344 | 3.02817 |
| 348 | 2.85914 | 2.75035 | 2.7957 | 2.91464 | 2.88732 |
| 360 | 2.92887 | 2.75035 | 2.86738 | 2.98404 | 2.88732 |
| 372 | 2.85914 | 2.82087 | 2.7957 | 2.84525 | 2.8169 |
| 384 | -- | -- | -- | -- | -- |
| 396 | 2.37099 | 2.39774 | 2.36559 | 2.35947 | 2.39437 |
| 408 | -- | -- | -- | -- | -- |
| 420 | 2.37099 | 2.46827 | 2.43728 | 2.35947 | 2.46479 |

## (4) Water-absorbing content:15.00 g/m2; Adhesive content:5.00 g/m2.

| Time(h) | Moisture content (%) | | | | |
| --- | --- | --- | --- | --- | --- |
|  | Weathered red-bed soil (%) of :0% | Weathered red-bed soil (%) of :1% | Weathered red-bed soil (%) of :2.5% | Weathered red-bed soil (%) of :5% | Weathered red-bed soil (%) of :10% |
| 0 | 50.20833 | 51.48014 | 50.45807 | 50.49575 | 50.83333 |
| 12 | -- | -- | -- | -- | -- |
| 24 | 36.94444 | 39.8556 | 40.45102 | 39.94334 | 39.93056 |
| 36 | -- | -- | -- | -- | -- |
| 48 | 30.06944 | 32.49097 | 33.05144 | 33.85269 | 34.16667 |
| 60 | 26.04167 | 28.15884 | 28.89359 | 30.24079 | 30.83333 |
| 72 | 22.08333 | 23.97112 | 25.15856 | 26.77054 | 27.56944 |
| 84 | 19.375 | 20.86643 | 22.48062 | 24.36261 | 25.41667 |
| 96 | 15.90278 | 17.11191 | 19.09796 | 21.10482 | 22.29167 |
| 108 | 13.19444 | 14.00722 | 16.27907 | 18.34278 | 19.72222 |
| 120 | 10.41667 | 10.54152 | 12.96688 | 15.01416 | 16.45833 |
| 132 | 8.95833 | 8.88087 | 10.92319 | 12.88952 | 14.44444 |
| 144 | 7.70833 | 7.36462 | 8.5976 | 10.41076 | 11.73611 |
| 156 | 6.73611 | 6.28159 | 7.39958 | 8.78187 | 9.93056 |
| 168 | 5.97222 | 5.41516 | 6.41297 | 7.5779 | 8.47222 |
| 180 | 5.41667 | 4.83755 | 5.70825 | 6.79887 | 7.56944 |
| 192 | 5 | 4.47653 | 5.14447 | 6.16147 | 6.80556 |
| 204 | 4.375 | 3.97112 | 4.65116 | 5.38244 | 6.04167 |
| 216 | 4.16667 | 3.68231 | 4.2988 | 5.09915 | 5.48611 |
| 228 | 3.80833 | 3.45884 | 3.87597 | 4.53258 | 3.75 |
| 240 | 3.68056 | 3.3213 | 3.66455 | 4.32011 | 4.58333 |
| 252 | -- | -- | -- | -- | -- |
| 264 | 3.26389 | 3.10469 | 3.24172 | 3.82436 | 3.88889 |
| 276 | 3.19444 | 2.96029 | 3.10078 | 3.68272 | 3.75 |
| 288 | 3.26389 | 3.03249 | 3.10078 | 3.6119 | 3.61111 |
| 300 | 2.98611 | 2.96029 | 2.88936 | 3.39943 | 3.33333 |
| 312 | 3.05556 | 2.96029 | 2.88936 | 3.32861 | 3.26389 |
| 324 | 2.91667 | 2.81588 | 2.81889 | 3.18697 | 3.125 |
| 336 | 2.84722 | 2.96029 | 2.74841 | 3.18697 | 3.05556 |
| 348 | 2.84722 | 2.96029 | 2.67794 | 3.04533 | 2.98611 |
| 360 | 2.91667 | 2.96029 | 2.81889 | 3.04533 | 2.98611 |
| 372 | 2.91667 | 2.96029 | 2.74841 | 2.9745 | 2.98611 |
| 384 | -- | -- | -- | -- | -- |
| 396 | 2.43056 | 2.45487 | 2.25511 | 2.6204 | 2.5 |
| 408 | -- | -- | -- | -- | -- |
| 420 | 2.5 | 2.52708 | 2.32558 | 2.6204 | 2.36111 |

## (5) Water-absorbing content:15.00 g/m2; Adhesive content:2.50 g/m2.

| Time(h) | Moisture content (%) | | | | |
| --- | --- | --- | --- | --- | --- |
|  | Weathered red-bed soil (%) of :0% | Weathered red-bed soil (%) of :1% | Weathered red-bed soil (%) of :2.5% | Weathered red-bed soil (%) of :5% | Weathered red-bed soil (%) of :10% |
| 0 | 50.81157 | 50 | 50.66573 | 50.59566 | 50.59482 |
| 12 | -- | -- | -- | -- | -- |
| 24 | 40.01411 | 39.67922 | 40.01402 | 38.76837 | 41.41556 |
| 36 | -- | -- | -- | -- | -- |
| 48 | 33.52152 | 33.33333 | 33.49685 | 33.17005 | 35.03854 |
| 60 | 29.21665 | 29.35844 | 29.64261 | 29.95101 | 31.39453 |
| 72 | 25.26464 | 25.59275 | 25.85844 | 26.87194 | 27.8206 |
| 84 | 22.51235 | 22.94282 | 23.47582 | 24.91253 | 25.50806 |
| 96 | 18.84263 | 19.3166 | 20.04205 | 22.18334 | 22.35459 |
| 108 | 15.87862 | 16.38773 | 17.30904 | 19.55151 | 19.80406 |
| 120 | 12.35004 | 13.04045 | 13.87526 | 15.97758 | 17.0049 |
| 132 | 10.4446 | 11.08787 | 11.9131 | 13.87526 | 15.18544 |
| 144 | 8.4686 | 9.13529 | 9.67064 | 11.14226 | 12.73618 |
| 156 | 7.26888 | 8.08926 | 8.40925 | 9.53048 | 10.91672 |
| 168 | 6.35145 | 7.11297 | 7.35809 | 8.12894 | 9.16725 |
| 180 | 5.78687 | 6.41562 | 6.65732 | 7.28802 | 8.18754 |
| 192 | 5.2223 | 5.92748 | 6.16678 | 6.7274 | 7.27782 |
| 204 | 4.58716 | 5.29986 | 5.60617 | 5.88648 | 6.29811 |
| 216 | 4.30487 | 4.95119 | 5.1857 | 5.46601 | 5.6683 |
| 228 | 3.95201 | 4.53278 | 4.62509 | 4.97547 | 5.03849 |
| 240 | 3.7403 | 4.32357 | 4.34478 | 4.62509 | 4.75857 |
| 252 | -- | -- | -- | -- | -- |
| 264 | 3.24629 | 3.69596 | 3.71409 | 3.85424 | 3.84885 |
| 276 | 3.10515 | 3.48675 | 3.50385 | 3.64401 | 3.63891 |
| 288 | 3.10515 | 3.48675 | 3.43378 | 3.57393 | 3.56893 |
| 300 | 2.96401 | 3.27755 | 3.22355 | 3.29362 | 3.28901 |
| 312 | 2.89344 | 3.13808 | 3.08339 | 3.15347 | 3.14906 |
| 324 | 2.82287 | 2.99861 | 3.01331 | 3.01331 | 3.0091 |
| 336 | 2.75229 | 2.99861 | 2.94324 | 2.94324 | 2.86914 |
| 348 | 2.61115 | 2.92887 | 2.87316 | 2.87316 | 2.72918 |
| 360 | 2.75229 | 2.92887 | 2.80308 | 2.87316 | 2.72918 |
| 372 | 2.75229 | 2.92887 | 2.80308 | 2.73301 | 2.72918 |
| 384 | -- | -- | -- | -- | -- |
| 396 | 2.32886 | 2.44073 | 2.38262 | 2.31254 | 2.23933 |
| 408 | -- | -- | -- | -- | -- |
| 420 | 2.32886 | 2.44073 | 2.38262 | 2.24247 | 2.30931 |

## (6) Water-absorbing content:0.00 g/m2; Adhesive content:0.00 g/m2.

| Time(h) | Moisture content (%) | | | | |
| --- | --- | --- | --- | --- | --- |
|  | Weathered red-bed soil (%) of :0% | Weathered red-bed soil (%) of :1% | Weathered red-bed soil (%) of :2.5% | Weathered red-bed soil (%) of :5% | Weathered red-bed soil (%) of :10% |
| 0 | 50.06553 | 50.45028 | 50.24493 | 50.87544 | 50.69444 |
| 12 | -- | -- | -- | -- | -- |
| 24 | 41.02564 | 39.91713 | 40.37789 | 41.11819 | 41.11111 |
| 36 | -- | -- | -- | -- | -- |
| 48 | 34.25926 | 33.08011 | 33.44997 | 35.03185 | 35.625 |
| 60 | 29.98575 | 29.07459 | 29.39118 | 31.21019 | 32.22222 |
| 72 | 25.78348 | 25.55249 | 25.61232 | 27.67162 | 28.95833 |
| 84 | 23.0057 | 23.0663 | 23.02309 | 25.12385 | 26.73611 |
| 96 | 19.37322 | 19.8895 | 19.73408 | 21.86837 | 23.75 |
| 108 | 16.38177 | 17.12707 | 17.14486 | 19.17905 | 21.25 |
| 120 | 12.82051 | 13.88122 | 13.92582 | 15.92357 | 18.19444 |
| 132 | 10.75499 | 11.67127 | 11.82645 | 13.80042 | 16.25 |
| 144 | 8.90313 | 9.04696 | 9.51714 | 11.11111 | 13.40278 |
| 156 | 7.76353 | 7.45856 | 8.25752 | 9.55414 | 11.25 |
| 168 | 6.83761 | 6.21547 | 7.06788 | 8.20948 | 9.30556 |
| 180 | 6.12536 | 5.31768 | 6.36809 | 7.36023 | 8.125 |
| 192 | 5.55556 | 4.83425 | 5.80826 | 6.72328 | 7.22222 |
| 204 | 5.05698 | 4.35083 | 5.24843 | 6.01557 | 6.25 |
| 216 | 4.70085 | 4.00552 | 4.82855 | 5.59094 | 5.625 |
| 228 | 4.2735 | 3.72928 | 4.3387 | 5.09554 | 5 |
| 240 | 4.13105 | 3.59116 | 4.12876 | 4.81246 | 4.72222 |
| 252 | -- | -- | -- | -- | -- |
| 264 | 3.7037 | 3.31492 | 3.63891 | 4.17551 | 3.88889 |
| 276 | 3.56125 | 3.1768 | 3.49895 | 4.03397 | 3.75 |
| 288 | 3.63248 | 3.24586 | 3.49895 | 3.9632 | 3.75 |
| 300 | 3.4188 | 3.03867 | 3.21903 | 3.75088 | 3.47222 |
| 312 | 3.34758 | 3.03867 | 3.21903 | 3.53857 | 3.40278 |
| 324 | 3.27635 | 2.96961 | 3.07908 | 3.39703 | 3.26389 |
| 336 | 3.27635 | 2.96961 | 3.14906 | 3.39703 | 3.26389 |
| 348 | 3.20513 | 2.96961 | 3.0091 | 3.32626 | 3.125 |
| 360 | 3.27635 | 2.96961 | 3.0091 | 3.32626 | 3.125 |
| 372 | 3.20513 | 3.03867 | 3.0091 | 3.25548 | 3.125 |
| 384 | -- | -- | -- | -- | -- |
| 396 | 2.77778 | 2.55525 | 2.58922 | 2.76008 | 2.63889 |
| 408 | -- | -- | -- | -- | -- |
| 420 | 2.849 | 2.62431 | 2.51924 | 2.76008 | 2.63889 |

# Fig 10. Moisture content of each sample with different amounts of additional weathered red-bed soil over 120 h (%)

| **materials** | **0.00/0.00 (g/m^2^)** | **15.00/2.50 (g/m^2^)** | **15.00/5.00 (g/m^2^)** | **30.00/2.50 (g/m^2^)** | **30.00/5.00 (g/m^2^)** | **60.00/10.00 (g/m^2^)** |
| --- | --- | --- | --- | --- | --- | --- |
| **0.00 (%)** | 10.41 | 11.95 | 12.32 | 12.34 | 13.20 | 13.09 |
| **1.00 (%)** | 10.55 | 11.45 | 13.02 | 13.31 | 13.86 | 14.55 |
| **2.50 (%)** | 12.94 | 13.17 | 13.85 | 14.24 | 15.64 | 16.04 |
| **5.00 (%)** | 14.71 | 14.98 | 15.92 | 16.27 | 16.72 | 17.73 |
| **10.00 (%)** | 15.17 | 16.40 | 16.92 | 18.83 | 18.12 | 19.64 |

# Shrinkage calculation of wax sealing method：

| Water-absorbing content/ Adhesive content (g/m^2^) | Weathered red-bed soil (%) | net weight (g) | Net weight and wax weight (g) | Weight in water (g) | Density (g/cm³) |
| --- | --- | --- | --- | --- | --- |
| 60/10 | 0 | 141.6 | 147.2 | 95.1 | 3.08 |
|  | 1 | 140.1 | 144.3 | 91.2 | 2.89 |
|  | 2.50 | 142 | 145.2 | 89.7 | 2.73 |
|  | 5 | 138.9 | 144.5 | 90.3 | 2.89 |
|  | 10 | 140.1 | 144.5 | 89.3 | 2.78 |
| 30/5 | 0 | 142.3 | 146.4 | 89.7 | 2.72 |
|  | 1 | 139.8 | 144.4 | 96 | 3.22 |
|  | 2.50 | 139.3 | 144.1 | 90.1 | 2.86 |
|  | 5 | 139.5 | 143.5 | 87.6 | 2.71 |
|  | 10 | 142 | 145.1 | 87.7 | 2.63 |
| 30/2.5 | 0 | 142.4 | 145.6 | 90 | 2.73 |
|  | 1 | 139.8 | 142.6 | 87.1 | 2.67 |
|  | 2.50 | 143 | 146.1 | 90.1 | 2.72 |
|  | 5 | 140.8 | 144.2 | 90.4 | 2.81 |
|  | 10 | 142.2 | 145.9 | 89.3 | 2.70 |
| 15/5 | 0 | 141.5 | 147.1 | 89.9 | 2.77 |
|  | 1 | 141.1 | 144.9 | 90 | 2.78 |
|  | 2.50 | 142.3 | 147.2 | 93.7 | 2.95 |
|  | 5 | 144 | 148.8 | 94.1 | 2.91 |
|  | 10 | 138 | 143.4 | 88.8 | 2.83 |
| 15/2.5 | 0 | 142.6 | 144.9 | 87.2 | 2.58 |
|  | 1 | 143.7 | 146.9 | 91.2 | 2.75 |
|  | 2.50 | 142.7 | 145.8 | 90.9 | 2.77 |
|  | 5 | 142.8 | 146.7 | 90.9 | 2.77 |
|  | 10 | 142.3 | 145.6 | 88.5 | 2.66 |
| 0/0 | 0 | 140.3 | 143.5 | 89.8 | 2.79 |
|  | 1 | 141.8 | 148.4 | 93.4 | 2.96 |
|  | 2.50 | 143 | 147 | 92.2 | 2.83 |
|  | 5 | 144.4 | 148.1 | 91.7 | 2.76 |
|  | 10 | 143.4 | 148.3 | 91.5 | 2.79 |

# Fig 11. Comparison of shrinkage rate (%)

| **materials** | **0.00/0.00 (g/m^2^)** | **15.00/2.50 (g/m^2^)** | **15.00/5.00 (g/m^2^)** | **30.00/2.50 (g/m^2^)** | **30.00/5.00 (g/m^2^)** | **60.00/10.00 (g/m^2^)** |
| --- | --- | --- | --- | --- | --- | --- |
| **0.00 (%)** | 28.48 | 29.83 | 30.23 | 30.36 | 31.29 | 31.03 |
| **1.00 (%)** | 28.56 | 29.65 | 29.98 | 29.35 | 30.56 | 30.13 |
| **2.50 (%)** | 28.60 | 28.20 | 28.22 | 28.32 | 28.86 | 29.67 |
| **5.00 (%)** | 27.71 | 27.48 | 28.57 | 28.14 | 26.78 | 29.38 |
| **10.00 (%)** | 26.63 | 27.32 | 26.25 | 28.17 | 26.63 | 25.61 |

# Fig 12. pH change at different amounts of added weathered red-bed soil

| **materials** | **0.00/0.00 (g/m^2^)** | **15.00/2.50 (g/m^2^)** | **15.00/5.00 (g/m^2^)** | **30.00/2.50 (g/m^2^)** | **30.00/5.00 (g/m^2^)** | **60.00/10.00 (g/m^2^)** |
| --- | --- | --- | --- | --- | --- | --- |
| **0.00 (%)** | 6.80 | 5.80 | 6.80 | 5.80 | 6.50 | 5.60 |
| **1.00 (%)** | 6.10 | 6.90 | 6.00 | 5.40 | 4.80 | 5.09 |
| **2.50 (%)** | 6.90 | 6.80 | 6.10 | 5.60 | 4.90 | 5.31 |
| **5.00 (%)** | 6.50 | 7.00 | 6.80 | 5.60 | 4.80 | 5.01 |
| **10.00 (%)** | 6.20 | 6.90 | 5.70 | 5.30 | 4.80 | 4.15 |

# Fig 13. Change in electrical conductivity at different amounts of added weathered red-bed soil (mS/cm)

| **materials** | **0.00/0.00 (g/m^2^)** | **15.00/2.50 (g/m^2^)** | **15.00/5.00 (g/m^2^)** | **30.00/2.50 (g/m^2^)** | **30.00/5.00 (g/m^2^)** | **60.00/10.00 (g/m^2^)** |
| --- | --- | --- | --- | --- | --- | --- |
| **0.00 (%)** | 0.024 | 0.044 | 0.044 | 0.066 | 0.072 | 0.054 |
| **1.00 (%)** | 0.036 | 0.054 | 0.051 | 0.076 | 0.078 | 0.066 |
| **2.50 (%)** | 0.030 | 0.066 | 0.066 | 0.066 | 0.078 | 0.096 |
| **5.00 (%)** | 0.042 | 0.054 | 0.072 | 0.084 | 0.084 | 0.086 |
| **10.00 (%)** | 0.064 | 0.074 | 0.084 | 0.096 | 0.09 | 0.096 |

# Expansion rate calculation：

| **Water-absorbing content:60.00 g/m2; Adhesive content:10.00 g/m2:** | | | | | |
| --- | --- | --- | --- | --- | --- |
| Weathered red-bed soil (%) | 0% | 1% | 2.50% | 5% | 10% |
| Initial | 4.682 | 1.622 | 4.888 | 6.082 | 4.649 |
| End | 8.568 | 4.935 | 9.231 | 9.012 | 8.921 |
| Expansion amount | 3.886 | 3.313 | 4.343 | 2.93 | 4.272 |
| Expansion rate (%) | 9.715 | 8.2825 | 10.8575 | 7.325 | 10.68 |
| **Water-absorbing content:30.00 g/m2; Adhesive content:5.00 g/m2:** | | | | | |
| Weathered red-bed soil (%) | 0% | 1% | 2.50% | 5% | 10% |
| Initial | 4.682 | 5.229 | 4.711 | 8.194 | 4.423 |
| End | 8.568 | 9.154 | 8.995 | 12.509 | 8.489 |
| Expansion amount | 3.886 | 3.925 | 4.284 | 4.315 | 4.066 |
| Expansion rate (%) | 9.715 | 9.8125 | 10.71 | 10.7875 | 10.165 |
| **Water-absorbing content:30.00 g/m2; Adhesive content:2.50 g/m2:** | | | | | |
| Weathered red-bed soil (%) | 0% | 1% | 2.50% | 5% | 10% |
| Initial | 4.928 | 8.593 | 8.889 | 3.757 | 7.861 |
| End | 8.619 | 12.345 | 12.817 | 7.578 | 12.133 |
| Expansion amount | 3.691 | 3.752 | 3.928 | 3.821 | 4.272 |
| Expansion rate (%) | 9.2275 | 9.38 | 9.82 | 9.5525 | 10.68 |
| **Water-absorbing content:15.00 g/m2; Adhesive content:5.00 g/m2:** | | | | | |
| Weathered red-bed soil (%) | 0% | 1% | 2.50% | 5% | 10% |
| Initial | 2.497 | 1.622 | 2.664 | 5.143 | 4.075 |
| End | 5.878 | 4.935 | 6.17 | 9.012 | 8.32 |
| Expansion amount | 3.381 | 3.313 | 3.506 | 3.869 | 4.245 |
| Expansion rate (%) | 8.4525 | 8.2825 | 8.765 | 9.6725 | 10.6125 |
| **Water-absorbing content:15.00 g/m2; Adhesive content:2.50 g/m2:** | | | | | |
| Weathered red-bed soil (%) | 0% | 1% | 2.50% | 5% | 10% |
| Initial | 4.809 | 2.223 | 2.784 | 6.082 | 6.097 |
| End | 7.912 | 5.601 | 6.257 | 9.012 | 10.169 |
| Expansion amount | 3.103 | 3.378 | 3.473 | 2.93 | 4.072 |
| Expansion rate (%) | 7.7575 | 8.445 | 8.6825 | 7.325 | 10.18 |
| **Water-absorbing content: 0.00 g/m2; Adhesive content:0.00 g/m2:** | | | | | |
| Weathered red-bed soil (%) | 0% | 1% | 2.50% | 5% | 10% |
| Initial | 4.779 | 2.932 | 6.082 | 9.637 | 5.005 |
| End | 7.681 | 5.652 | 9.035 | 12.809 | 8.361 |
| Expansion amount | 2.902 | 2.72 | 2.953 | 3.172 | 3.356 |
| Expansion rate (%) | 7.255 | 6.8 | 7.3825 | 7.93 | 8.39 |

# Fig 14. Comparison of the expansion rate at different levels of added weathered red-bed soil (%)

| **materials** | **0.00/0.00 (g/m^2^)** | **15.00/2.50 (g/m^2^)** | **15.00/5.00 (g/m^2^)** | **30.00/2.50 (g/m^2^)** | **30.00/5.00 (g/m^2^)** | **60.00/10.00 (g/m^2^)** |
| --- | --- | --- | --- | --- | --- | --- |
| **0.00 (%)** | 7.26 | 7.76 | 8.45 | 9.23 | 9.72 | 10.58 |
| **1.00 (%)** | 6.80 | 8.45 | 8.28 | 9.38 | 9.81 | 10.70 |
| **2.50 (%)** | 7.39 | 8.68 | 8.77 | 9.82 | 10.71 | 10.86 |
| **5.00 (%)** | 7.93 | 7.33 | 9.67 | 9.55 | 10.79 | 11.38 |
| **10.00 (%)** | 8.39 | 10.18 | 10.61 | 10.68 | 10.17 | 11.34 |

# permeability computation

## (1) Water-absorbing content:60.00 g/m^2^; Adhesive content:10.00 g/m^2^; Weathered red-bed soil 0.00%:

| Start: t_1_ (min) | Finish: t_2_ | Duration: t | To start the water head: h1(cm) | End the water head:h_2_ (cm) | $2.3\frac{a}{A}\frac{L}{t}$ | $lg\frac{h_{1}}{h_{2}}$ | Permeability coefficient at water temperature T °C:k_T_ (cm/s) | Water temperature (℃) | Correction coefficient | Permeability coefficient: k_20_ (cm/s) | Average permeability coefficient:k_20_ (cm/s) |
| --- | --- | --- | --- | --- | --- | --- | --- | --- | --- | --- | --- |
| 1 | 2 | 3 | 4 | 5 | 6 | 7 | 8 | 9 | 10 | 11 | 12 |
|  |  | (2)-(1) |  |  |  |  | (6)×(7) |  |  | (8)×(10) | $\frac{\sum(11)}{n}$ |
| 0 | 282 | 282 | 1900 | 525 | 0.0005674 | 0.5587 | 0.000317 | 27 | 0.85 | 0.0002695 | 0.00022 |
| 0 | 311 | 311 | 1631 | 478 | 0.0005145 | 0.533 | 0.0002742 | 27 | 0.85 | 0.0002331 |  |
| 0 | 430 | 430 | 1616 | 335 | 0.0003721 | 0.6834 | 0.0002543 | 27 | 0.85 | 0.0002162 |  |
| 0 | 309 | 309 | 1624 | 570 | 0.0005178 | 0.4548 | 0.0002355 | 27 | 0.85 | 0.0002002 |  |
| 0 | 513 | 513 | 1622 | 296 | 0.0003119 | 0.7388 | 0.0002304 | 27 | 0.85 | 0.0001958 |  |
| 0 | 377 | 377 | 1742 | 540 | 0.0004244 | 0.5087 | 0.0002159 | 27 | 0.85 | 0.0001835 |  |
| 0 | 349 | 349 | 1642 | 568 | 0.0004585 | 0.461 | 0.0002114 | 27 | 0.85 | 0.0001797 |  |

**Record of water head:**

| Times | Hour | Minute | Second | Start water head | Ended  water head | correction number | Total seconds | Corrects start water head | Corrects ended water head |
| --- | --- | --- | --- | --- | --- | --- | --- | --- | --- |
| 1 | 0 | 0 | 0 | 1935 |  | -35 | 0 | 1900 |  |
| 1 | 0 | 4 | 42 |  | 560 | -35 | 282 |  | 525 |
| 2 | 0 | 0 | 0 | 1666 |  | -35 | 0 | 1631 |  |
| 2 | 0 | 5 | 11 |  | 513 | -35 | 311 |  | 478 |
| 3 | 0 | 0 | 0 | 1651 |  | -35 | 0 | 1616 |  |
| 3 | 0 | 7 | 10 |  | 370 | -35 | 430 |  | 335 |
| 4 | 0 | 0 | 0 | 1659 |  | -35 | 0 | 1624 |  |
| 4 | 0 | 5 | 9 |  | 605 | -35 | 309 |  | 570 |
| 5 | 0 | 0 | 0 | 1657 |  | -35 | 0 | 1622 |  |
| 5 | 0 | 8 | 33 |  | 331 | -35 | 513 |  | 296 |
| 6 | 0 | 0 | 0 | 1777 |  | -35 | 0 | 1742 |  |
| 6 | 0 | 6 | 17 |  | 575 | -35 | 377 |  | 540 |
| 7 | 0 | 0 | 0 | 1677 |  | -35 | 0 | 1642 |  |
| 7 | 0 | 5 | 49 |  | 603 | -35 | 349 |  | 568 |

## (2) Water-absorbing content:60.00 g/m^2^; Adhesive content:10.00 g/m^2^; Weathered red-bed soil 1.00%:

| Start: t_1_ (min) | Finish: t_2_ | Duration: t | To start the water head: h1(cm) | End the water head:h_2_ (cm) | $2.3\frac{a}{A}\frac{L}{t}$ | $lg\frac{h_{1}}{h_{2}}$ | Permeability coefficient at water temperature T °C:k_T_ (cm/s) | Water temperature (℃) | Correction coefficient | Permeability coefficient: k_20_ (cm/s) | Average permeability coefficient:k_20_ (cm/s) |
| --- | --- | --- | --- | --- | --- | --- | --- | --- | --- | --- | --- |
| 1 | 2 | 3 | 4 | 5 | 6 | 7 | 8 | 9 | 10 | 11 | 12 |
|  |  | (2)-(1) |  |  | $2.3\frac{a}{A}\frac{L}{(3)}$ | $lg\frac{(4)}{(5)}$ | (6)×(7) |  |  | (8)×(10) | $\frac{\sum(11)}{n}$ |
| 0 | 606 | 606 | 1970 | 564 | 0.000264 | 0.5428 | 0.0001433 | 26 | 0.87 | 0.0001247 | 0.00012 |
| 0 | 694 | 694 | 1829 | 438 | 0.0002305 | 0.6207 | 0.0001431 | 26 | 0.87 | 0.0001245 |  |
| 0 | 582 | 582 | 1670 | 507 | 0.0002749 | 0.5177 | 0.0001423 | 26 | 0.87 | 0.0001238 |  |
| 0 | 601 | 601 | 1759 | 531 | 0.0002662 | 0.5198 | 0.0001384 | 26 | 0.87 | 0.0001204 |  |
| 0 | 1142 | 1142 | 1752 | 186 | 0.0001401 | 0.974 | 0.0001365 | 26 | 0.87 | 0.0001188 |  |
| 0 | 552 | 552 | 1770 | 617 | 0.0002899 | 0.4577 | 0.0001327 | 26 | 0.87 | 0.0001154 |  |
| 0 | 598 | 598 | 1789 | 573 | 0.0002676 | 0.4945 | 0.0001323 | 26 | 0.87 | 0.0001151 |  |

**Record of water head:**

| Times | Hour | Minute | Second | Start water head | Ended  water head | correction number | Total seconds | Corrects start water head | Corrects ended water head |
| --- | --- | --- | --- | --- | --- | --- | --- | --- | --- |
| 1 | 0 | 0 | 0 | 2000 |  | -30 | 0 | 1970 |  |
| 1 | 0 | 10 | 6 |  | 594 | -30 | 606 |  | 564 |
| 2 | 0 | 0 | 0 | 1859 |  | -30 | 0 | 1829 |  |
| 2 | 0 | 11 | 34 |  | 468 | -30 | 694 |  | 438 |
| 3 | 0 | 0 | 0 | 1700 |  | -30 | 0 | 1670 |  |
| 3 | 0 | 9 | 42 |  | 537 | -30 | 582 |  | 507 |
| 4 | 0 | 0 | 0 | 1789 |  | -30 | 0 | 1759 |  |
| 4 | 0 | 10 | 1 |  | 561 | -30 | 601 |  | 531 |
| 5 | 0 | 0 | 0 | 1782 |  | -30 | 0 | 1752 |  |
| 5 | 0 | 19 | 2 |  | 216 | -30 | 1142 |  | 186 |
| 6 | 0 | 0 | 0 | 1800 |  | -30 | 0 | 1770 |  |
| 6 | 0 | 9 | 12 |  | 647 | -30 | 552 |  | 617 |
| 7 | 0 | 0 | 0 | 1819 |  | -30 | 0 | 1789 |  |
| 7 | 0 | 9 | 58 |  | 603 | -30 | 598 |  | 573 |

## (3) Water-absorbing content:60.00 g/m^2^; Adhesive content:10.00 g/m^2^; Weathered red-bed soil 2.50%:

| Start: t_1_ (min) | Finish: t_2_ | Duration: t | To start the water head: h1(cm) | End the water head:h_2_ (cm) | $2.3\frac{a}{A}\frac{L}{t}$ | $lg\frac{h_{1}}{h_{2}}$ | Permeability coefficient at water temperature T °C:k_T_ (cm/s) | Water temperature (℃) | Correction coefficient | Permeability coefficient: k_20_ (cm/s) | Average permeability coefficient:k_20_ (cm/s) |
| --- | --- | --- | --- | --- | --- | --- | --- | --- | --- | --- | --- |
| 1 | 2 | 3 | 4 | 5 | 6 | 7 | 8 | 9 | 10 | 11 | 12 |
|  |  | (2)-(1) |  |  | $2.3\frac{a}{A}\frac{L}{(3)}$ | $lg\frac{(4)}{(5)}$ | (6)×(7) |  |  | (8)×(10) | $\frac{\sum(11)}{n}$ |
| 0 | 930 | 930 | 1774 | 162 | 0.000172 | 1.0394 | 0.0001788 | 27 | 0.85 | 0.000152 | 0.0001357 |
| 0 | 580 | 580 | 1859 | 451 | 0.0002759 | 0.6151 | 0.0001697 | 27 | 0.85 | 0.0001442 |  |
| 0 | 581 | 581 | 1873 | 486 | 0.0002754 | 0.5859 | 0.0001614 | 27 | 0.85 | 0.0001372 |  |
| 0 | 538 | 538 | 1652 | 496 | 0.0002974 | 0.5224 | 0.0001554 | 27 | 0.85 | 0.0001321 |  |
| 0 | 518 | 518 | 1876 | 617 | 0.0003089 | 0.4829 | 0.0001492 | 27 | 0.85 | 0.0001268 |  |
| 0 | 445 | 445 | 1684 | 673 | 0.0003596 | 0.3983 | 0.0001432 | 27 | 0.85 | 0.0001217 |  |
| 0 | 703 | 703 | 1785 | 455 | 0.0002276 | 0.5936 | 0.0001351 | 27 |  |  |  |

**Record of water head:**

| Times | Hour | Minute | Second | Start water head | Ended  water head | correction number | Total seconds | Corrects start water head | Corrects ended water head |
| --- | --- | --- | --- | --- | --- | --- | --- | --- | --- |
| 1 | 0 | 0 | 0 | 1814 |  | -40 | 0 | 1774 |  |
| 1 | 0 | 15 | 30 |  | 202 | -40 | 930 |  | 162 |
| 2 | 0 | 0 | 0 | 1899 |  | -40 | 0 | 1859 |  |
| 2 | 0 | 9 | 40 |  | 491 | -40 | 580 |  | 451 |
| 3 | 0 | 0 | 0 | 1913 |  | -40 | 0 | 1873 |  |
| 3 | 0 | 9 | 41 |  | 526 | -40 | 581 |  | 486 |
| 4 | 0 | 0 | 0 | 1692 |  | -40 | 0 | 1652 |  |
| 4 | 0 | 8 | 58 |  | 536 | -40 | 538 |  | 496 |
| 5 | 0 | 0 | 0 | 1916 |  | -40 | 0 | 1876 |  |
| 5 | 0 | 8 | 38 |  | 657 | -40 | 518 |  | 617 |
| 6 | 0 | 0 | 0 | 1724 |  | -40 | 0 | 1684 |  |
| 6 | 0 | 7 | 25 |  | 713 | -40 | 445 |  | 673 |
| 7 | 0 | 0 | 0 | 1825 |  | -40 | 0 | 1785 |  |
| 7 | 0 | 11 | 43 |  | 495 | -40 | 703 |  | 455 |

## (4) Water-absorbing content:60.00 g/m^2^; Adhesive content:10.00 g/m^2^; Weathered red-bed soil 5.00%:

| Start: t_1_ (min) | Finish: t_2_ | Duration: t | To start the water head: h1(cm) | End the water head:h_2_ (cm) | $2.3\frac{a}{A}\frac{L}{t}$ | $lg\frac{h_{1}}{h_{2}}$ | Permeability coefficient at water temperature T °C:k_T_ (cm/s) | Water temperature (℃) | Correction coefficient | Permeability coefficient: k_20_ (cm/s) | Average permeability coefficient:k_20_ (cm/s) |
| --- | --- | --- | --- | --- | --- | --- | --- | --- | --- | --- | --- |
| 1 | 2 | 3 | 4 | 5 | 6 | 7 | 8 | 9 | 10 | 11 | 12 |
|  |  | (2)-(1) |  |  | $2.3\frac{a}{A}\frac{L}{(3)}$ | $lg\frac{(4)}{(5)}$ | (6)×(7) |  |  | (8)×(10) | $\frac{\sum(11)}{n}$ |
| 0 | 1353 | 1353 | 1710 | 360 | 0.0001183 | 0.6767 | 0.0000801 | 26 | 0.87 | 0.0000697 | 0.0000662 |
| 0 | 751 | 751 | 1455 | 631 | 0.000213 | 0.3628 | 0.0000773 | 26 | 0.87 | 0.0000673 |  |
| 0 | 964 | 964 | 1678 | 576 | 0.000166 | 0.4644 | 0.0000771 | 26 | 0.87 | 0.0000671 |  |
| 0 | 1144 | 1144 | 1768 | 509 | 0.0001399 | 0.5403 | 0.0000756 | 26 | 0.87 | 0.0000658 |  |
| 0 | 1223 | 1223 | 1827 | 492 | 0.0001308 | 0.5698 | 0.0000745 | 26 | 0.87 | 0.0000648 |  |
| 0 | 997 | 997 | 1821 | 650 | 0.0001605 | 0.4474 | 0.0000718 | 26 | 0.87 | 0.0000625 |  |
| 0 | 4641 | 4641 | 2135 | 805 | 0.0000345 | 0.4236 | 0.0000146 | 17 |  |  |  |

**Record of water head:**

| Times | Hour | Minute | Second | Start water head | Ended  water head | correction number | Total seconds | Corrects start water head | Corrects ended water head |
| --- | --- | --- | --- | --- | --- | --- | --- | --- | --- |
| 1 | 0 | 0 | 0 | 1745 |  | -35 | 0 | 1710 |  |
| 1 | 0 | 22 | 33 |  | 395 | -35 | 1353 |  | 360 |
| 2 | 0 | 0 | 0 | 1490 |  | -35 | 0 | 1455 |  |
| 2 | 0 | 12 | 31 |  | 666 | -35 | 751 |  | 631 |
| 3 | 0 | 0 | 0 | 1713 |  | -35 | 0 | 1678 |  |
| 3 | 0 | 16 | 4 |  | 611 | -35 | 964 |  | 576 |
| 4 | 0 | 0 | 0 | 1803 |  | -35 | 0 | 1768 |  |
| 4 | 0 | 19 | 4 |  | 544 | -35 | 1144 |  | 509 |
| 5 | 0 | 0 | 0 | 1862 |  | -35 | 0 | 1827 |  |
| 5 | 0 | 20 | 23 |  | 527 | -35 | 1223 |  | 492 |
| 6 | 0 | 0 | 0 | 1856 |  | -35 | 0 | 1821 |  |
| 6 | 0 | 16 | 37 |  | 685 | -35 | 997 |  | 650 |
| 7 | 0 | 0 | 0 | 1705 |  | 430 | 0 | 2135 |  |
| 7 | 1 | 17 | 21 |  | 375 | 430 | 4641 |  | 805 |

## (5) Water-absorbing content:60.00 g/m^2^; Adhesive content:10.00 g/m^2^; Weathered red-bed soil 10.00%:

| Start: t_1_ (min) | Finish: t_2_ | Duration: t | To start the water head: h1(cm) | End the water head:h_2_ (cm) | $2.3\frac{a}{A}\frac{L}{t}$ | $lg\frac{h_{1}}{h_{2}}$ | Permeability coefficient at water temperature T °C:k_T_ (cm/s) | Water temperature (℃) | Correction coefficient | Permeability coefficient: k_20_ (cm/s) | Average permeability coefficient:k_20_ (cm/s) |
| --- | --- | --- | --- | --- | --- | --- | --- | --- | --- | --- | --- |
| 1 | 2 | 3 | 4 | 5 | 6 | 7 | 8 | 9 | 10 | 11 | 12 |
|  |  | (2)-(1) |  |  | $2.3\frac{a}{A}\frac{L}{(3)}$ | $lg\frac{(4)}{(5)}$ | (6)×(7) |  |  | (8)×(10) | $\frac{\sum(11)}{n}$ |
| 0 | 1353 | 1353 | 1655 | 765 | 0.0001183 | 0.3345 | 0.0000396 | 26 | 0.87 | 0.0000345 | 0.0000314 |
| 0 | 1797 | 1797 | 1760 | 659 | 0.000089 | 0.4266 | 0.000038 | 26 | 0.87 | 0.0000331 |  |
| 0 | 1787 | 1787 | 1790 | 706 | 0.0000895 | 0.404 | 0.0000362 | 26 | 0.87 | 0.0000315 |  |
| 0 | 1692 | 1692 | 1865 | 800 | 0.0000946 | 0.3674 | 0.0000348 | 26 | 0.87 | 0.0000303 |  |
| 0 | 907 | 907 | 1675 | 1070 | 0.0001764 | 0.1946 | 0.0000343 | 26 | 0.87 | 0.0000298 |  |
| 0 | 1619 | 1619 | 1858 | 856 | 0.0000988 | 0.3366 | 0.0000333 | 26 | 0.87 | 0.000029 |  |
| 0 | 4641 | 4641 | 2135 | 805 | 0.0000345 | 0.4236 | 0.0000146 | 17 |  |  |  |

**Record of water head:**

| Times | Hour | Minute | Second | Start water head | Ended  water head | correction number | Total seconds | Corrects start water head | Corrects ended water head |
| --- | --- | --- | --- | --- | --- | --- | --- | --- | --- |
| 1 | 0 | 0 | 0 | 1695 |  | -40 | 0 | 1655 |  |
| 1 | 0 | 22 | 33 |  | 805 | -40 | 1353 |  | 765 |
| 2 | 0 | 0 | 0 | 1800 |  | -40 | 0 | 1760 |  |
| 2 | 0 | 29 | 57 |  | 699 | -40 | 1797 |  | 659 |
| 3 | 0 | 0 | 0 | 1830 |  | -40 | 0 | 1790 |  |
| 3 | 0 | 29 | 47 |  | 746 | -40 | 1787 |  | 706 |
| 4 | 0 | 0 | 0 | 1905 |  | -40 | 0 | 1865 |  |
| 4 | 0 | 28 | 12 |  | 840 | -40 | 1692 |  | 800 |
| 5 | 0 | 0 | 0 | 1715 |  | -40 | 0 | 1675 |  |
| 5 | 0 | 15 | 7 |  | 1110 | -40 | 907 |  | 1070 |
| 6 | 0 | 0 | 0 | 1898 |  | -40 | 0 | 1858 |  |
| 6 | 0 | 26 | 59 |  | 896 | -40 | 1619 |  | 856 |
| 7 | 0 | 0 | 0 | 1705 |  | 430 | 0 | 2135 |  |
| 7 | 1 | 17 | 21 |  | 375 | 430 | 4641 |  | 805 |

## (6) Water-absorbing content:30.00 g/m^2^; Adhesive content:5.00 g/m^2^; Weathered red-bed soil 0.00%:

| Start: t_1_ (min) | Finish: t_2_ | Duration: t | To start the water head: h1(cm) | End the water head:h_2_ (cm) | $2.3\frac{a}{A}\frac{L}{t}$ | $lg\frac{h_{1}}{h_{2}}$ | Permeability coefficient at water temperature T °C:k_T_ (cm/s) | Water temperature (℃) | Correction coefficient | Permeability coefficient: k_20_ (cm/s) | Average permeability coefficient:k_20_ (cm/s) |
| --- | --- | --- | --- | --- | --- | --- | --- | --- | --- | --- | --- |
| 1 | 2 | 3 | 4 | 5 | 6 | 7 | 8 | 9 | 10 | 11 | 12 |
|  |  | (2)-(1) |  |  | $2.3\frac{a}{A}\frac{L}{(3)}$ | $lg\frac{(4)}{(5)}$ | (6)×(7) |  |  | (8)×(10) | $\frac{\sum(11)}{n}$ |
| 0 | 1008 | 1008 | 1620 | 701 | 0.0001587 | 0.3636 | 0.0000577 | 26 | 0.87 | 0.0000502 | 4.3E-05 |
| 0 | 1144 | 1144 | 1681 | 717 | 0.0001399 | 0.37 | 0.0000518 | 26 | 0.87 | 0.0000451 |  |
| 0 | 1520 | 1520 | 1750 | 618 | 0.0001053 | 0.452 | 0.0000476 | 26 | 0.87 | 0.0000414 |  |
| 0 | 1469 | 1469 | 1783 | 629 | 0.0001089 | 0.4518 | 0.0000492 | 26 | 0.87 | 0.0000428 |  |
| 0 | 1492 | 1492 | 1828 | 672 | 0.0001072 | 0.4346 | 0.0000466 | 26 | 0.87 | 0.0000405 |  |
| 0 | 1337 | 1337 | 1805 | 755 | 0.0001197 | 0.3785 | 0.0000453 | 26 | 0.87 | 0.0000394 |  |

**Record of water head:**

| Times | Hour | Minute | Second | Start water head | Ended  water head | correction number | Total seconds | Corrects start water head | Corrects ended water head |
| --- | --- | --- | --- | --- | --- | --- | --- | --- | --- |
| 1 | 0 | 0 | 0 | 1660 |  | -40 | 0 | 1620 |  |
| 1 | 0 | 16 | 48 |  | 741 | -40 | 1008 |  | 701 |
| 2 | 0 | 0 | 0 | 1721 |  | -40 | 0 | 1681 |  |
| 2 | 0 | 19 | 4 |  | 757 | -40 | 1144 |  | 717 |
| 3 | 0 | 0 | 0 | 1790 |  | -40 | 0 | 1750 |  |
| 3 | 0 | 25 | 20 |  | 658 | -40 | 1520 |  | 618 |
| 4 | 0 | 0 | 0 | 1823 |  | -40 | 0 | 1783 |  |
| 4 | 0 | 24 | 29 |  | 669 | -40 | 1469 |  | 629 |
| 5 | 0 | 0 | 0 | 1868 |  | -40 | 0 | 1828 |  |
| 5 | 0 | 24 | 52 |  | 712 | -40 | 1492 |  | 672 |
| 6 | 0 | 0 | 0 | 1845 |  | -40 | 0 | 1805 |  |
| 6 | 0 | 22 | 17 |  | 795 | -40 | 1337 |  | 755 |

## (7) Water-absorbing content:30.00 g/m^2^; Adhesive content:5.00 g/m^2^; Weathered red-bed soil 1.00%:

| Start: t_1_ (min) | Finish: t_2_ | Duration: t | To start the water head: h1(cm) | End the water head:h_2_ (cm) | $2.3\frac{a}{A}\frac{L}{t}$ | $lg\frac{h_{1}}{h_{2}}$ | Permeability coefficient at water temperature T °C:k_T_ (cm/s) | Water temperature (℃) | Correction coefficient | Permeability coefficient: k_20_ (cm/s) | Average permeability coefficient:k_20_ (cm/s) |
| --- | --- | --- | --- | --- | --- | --- | --- | --- | --- | --- | --- |
| 1 | 2 | 3 | 4 | 5 | 6 | 7 | 8 | 9 | 10 | 11 | 12 |
|  |  | (2)-(1) |  |  | $2.3\frac{a}{A}\frac{L}{(3)}$ | $lg\frac{(4)}{(5)}$ | (6)×(7) |  |  | (8)×(10) | $\frac{\sum(11)}{n}$ |
| 0 | 295 | 295 | 1836 | 610 | 0.0005424 | 0.4786 | 0.0002596 | 27 | 0.85 | 0.0002207 | 0.000183 |
| 0 | 312 | 312 | 1690 | 601 | 0.0005128 | 0.449 | 0.0002302 | 27 | 0.85 | 0.0001957 |  |
| 0 | 429 | 429 | 1667 | 443 | 0.000373 | 0.5755 | 0.0002147 | 27 | 0.85 | 0.0001825 |  |
| 0 | 309 | 309 | 1610 | 656 | 0.0005178 | 0.3892 | 0.0002015 | 27 | 0.85 | 0.0001713 |  |
| 0 | 513 | 513 | 1640 | 380 | 0.0003119 | 0.6351 | 0.0001981 | 27 | 0.85 | 0.0001684 |  |
| 0 | 378 | 378 | 1764 | 631 | 0.0004233 | 0.4465 | 0.000189 | 27 | 0.85 | 0.0001607 |  |
| 0 | 345 | 345 | 1585 | 629 | 0.0004638 | 0.4014 | 0.0001862 | 27 | 0.85 | 0.0001583 |  |

**Record of water head:**

| Times | Hour | Minute | Second | Start water head | Ended  water head | correction number | Total seconds | Corrects start water head | Corrects ended water head |
| --- | --- | --- | --- | --- | --- | --- | --- | --- | --- |
| 1 | 0 | 0 | 0 | 1871 |  | -35 | 0 | 1836 |  |
| 1 | 0 | 4 | 55 |  | 645 | -35 | 295 |  | 610 |
| 2 | 0 | 0 | 0 | 1725 |  | -35 | 0 | 1690 |  |
| 2 | 0 | 5 | 12 |  | 636 | -35 | 312 |  | 601 |
| 3 | 0 | 0 | 0 | 1702 |  | -35 | 0 | 1667 |  |
| 3 | 0 | 7 | 9 |  | 478 | -35 | 429 |  | 443 |
| 4 | 0 | 0 | 0 | 1645 |  | -35 | 0 | 1610 |  |
| 4 | 0 | 5 | 9 |  | 691 | -35 | 309 |  | 656 |
| 5 | 0 | 0 | 0 | 1675 |  | -35 | 0 | 1640 |  |
| 5 | 0 | 8 | 33 |  | 415 | -35 | 513 |  | 380 |
| 6 | 0 | 0 | 0 | 1799 |  | -35 | 0 | 1764 |  |
| 6 | 0 | 6 | 18 |  | 666 | -35 | 378 |  | 631 |
| 7 | 0 | 0 | 0 | 1620 |  | -35 | 0 | 1585 |  |
| 7 | 0 | 5 | 45 |  | 664 | -35 | 345 |  | 629 |

## (8) Water-absorbing content:30.00 g/m^2^; Adhesive content:5.00 g/m^2^; Weathered red-bed soil 2.50%:

| Start: t_1_ (min) | Finish: t_2_ | Duration: t | To start the water head: h1(cm) | End the water head:h_2_ (cm) | $2.3\frac{a}{A}\frac{L}{t}$ | $lg\frac{h_{1}}{h_{2}}$ | Permeability coefficient at water temperature T °C:k_T_ (cm/s) | Water temperature (℃) | Correction coefficient | Permeability coefficient: k_20_ (cm/s) | Average permeability coefficient:k_20_ (cm/s) |
| --- | --- | --- | --- | --- | --- | --- | --- | --- | --- | --- | --- |
| 1 | 2 | 3 | 4 | 5 | 6 | 7 | 8 | 9 | 10 | 11 | 12 |
|  |  | (2)-(1) |  |  | $2.3\frac{a}{A}\frac{L}{(3)}$ | $lg\frac{(4)}{(5)}$ | (6)×(7) |  |  | (8)×(10) | $\frac{\sum(11)}{n}$ |
| 0 | 1035 | 1035 | 1760 | 883 | 0.0001546 | 0.2989 | 0.0000462 | 27 | 0.85 | 0.0000393 | 0.0000378 |
| 0 | 1159 | 1159 | 1725 | 804 | 0.0001381 | 0.3315 | 0.0000458 | 27 | 0.85 | 0.0000389 |  |
| 0 | 1281 | 1281 | 1705 | 742 | 0.0001249 | 0.3613 | 0.0000451 | 27 | 0.85 | 0.0000383 |  |
| 0 | 1238 | 1238 | 1786 | 813 | 0.0001292 | 0.3424 | 0.0000442 | 27 | 0.85 | 0.0000376 |  |
| 0 | 1611 | 1611 | 1825 | 663 | 0.0000993 | 0.4397 | 0.0000437 | 27 | 0.85 | 0.0000371 |  |
| 0 | 1189 | 1189 | 1970 | 958 | 0.0001346 | 0.3131 | 0.0000421 | 27 | 0.85 | 0.0000358 |  |
| 0 | 4641 | 4641 | 2135 | 805 | 0.0000345 | 0.4236 | 0.0000146 | 17 |  |  |  |

**Record of water head:**

| Times | Hour | Minute | Second | Start water head | Ended  water head | correction number | Total seconds | Corrects start water head | Corrects ended water head |
| --- | --- | --- | --- | --- | --- | --- | --- | --- | --- |
| 1 | 0 | 0 | 0 | 1790 |  | -30 | 0 | 1760 |  |
| 1 | 0 | 17 | 15 |  | 913 | -30 | 1035 |  | 883 |
| 2 | 0 | 0 | 0 | 1755 |  | -30 | 0 | 1725 |  |
| 2 | 0 | 19 | 19 |  | 834 | -30 | 1159 |  | 804 |
| 3 | 0 | 0 | 0 | 1735 |  | -30 | 0 | 1705 |  |
| 3 | 0 | 21 | 21 |  | 772 | -30 | 1281 |  | 742 |
| 4 | 0 | 0 | 0 | 1816 |  | -30 | 0 | 1786 |  |
| 4 | 0 | 20 | 38 |  | 843 | -30 | 1238 |  | 813 |
| 5 | 0 | 0 | 0 | 1855 |  | -30 | 0 | 1825 |  |
| 5 | 0 | 26 | 51 |  | 693 | -30 | 1611 |  | 663 |
| 6 | 0 | 0 | 0 | 2000 |  | -30 | 0 | 1970 |  |
| 6 | 0 | 19 | 49 |  | 988 | -30 | 1189 |  | 958 |
| 7 | 0 | 0 | 0 | 1705 |  | 430 | 0 | 2135 |  |
| 7 | 1 | 17 | 21 |  | 375 | 430 | 4641 |  | 805 |

## (9) Water-absorbing content:30.00 g/m^2^; Adhesive content:5.00 g/m^2^; Weathered red-bed soil 0.00%:

| Start: t_1_ (min) | Finish: t_2_ | Duration: t | To start the water head: h1(cm) | End the water head:h_2_ (cm) | $2.3\frac{a}{A}\frac{L}{t}$ | $lg\frac{h_{1}}{h_{2}}$ | Permeability coefficient at water temperature T °C:k_T_ (cm/s) | Water temperature (℃) | Correction coefficient | Permeability coefficient: k_20_ (cm/s) | Average permeability coefficient:k_20_ (cm/s) |
| --- | --- | --- | --- | --- | --- | --- | --- | --- | --- | --- | --- |
| 1 | 2 | 3 | 4 | 5 | 6 | 7 | 8 | 9 | 10 | 11 | 12 |
|  |  | (2)-(1) |  |  | $2.3\frac{a}{A}\frac{L}{(3)}$ | $lg\frac{(4)}{(5)}$ | (6)×(7) |  |  | (8)×(10) | $\frac{\sum(11)}{n}$ |
|  |  | (2)-(1) |  |  |  |  | (6)×(7) |  |  | (8)×(10) | 0.000058 |
|  |  |  |  |  |  |  |  |  |  |  |  |
| 0 | 2139 | 2139 | 1865 | 172 | 0.0000748 | 1.035 | 0.0000774 | 27 | 0.85 | 0.0000658 |  |
| 0 | 911 | 911 | 1612 | 630 | 0.0001756 | 0.408 | 0.0000716 | 27 | 0.85 | 0.0000609 |  |
| 0 | 1091 | 1091 | 1837 | 689 | 0.0001467 | 0.4259 | 0.0000625 | 27 | 0.85 | 0.0000531 |  |
| 0 | 894 | 894 | 1588 | 660 | 0.0001790 | 0.382 | 0.0000684 | 27 | 0.85 | 0.0000581 |  |
| 0 | 1125 | 1125 | 1884 | 649 | 0.0001422 | 0.4628 | 0.0000658 | 27 | 0.85 | 0.0000559 |  |

**Record of water head:**

| Times | Hour | Minute | Second | Start water head | Ended  water head | correction number | Total seconds | Corrects start water head | Corrects ended water head |
| --- | --- | --- | --- | --- | --- | --- | --- | --- | --- |
| 1 | 0 | 0 | 0 | 1905 |  | -40 | 0 | 1865 |  |
| 1 | 0 | 35 | 39 |  | 212 | -40 | 2139 |  | 172 |
| 2 | 0 | 0 | 0 | 1652 |  | -40 | 0 | 1612 |  |
| 2 | 0 | 15 | 11 |  | 670 | -40 | 911 |  | 630 |
| 3 | 0 | 0 | 0 | 1877 |  | -40 | 0 | 1837 |  |
| 3 | 0 | 18 | 11 |  | 729 | -40 | 1091 |  | 689 |
| 4 | 0 | 0 | 0 | 1628 |  | -40 | 0 | 1588 |  |
| 4 | 0 | 14 | 54 |  | 700 | -40 | 894 |  | 660 |
| 5 | 0 |  | 0 | 1924 |  | -40 | 0 | 1884 |  |
| 5 | 0 | 18 | 45 |  | 689 | -40 | 1125 |  | 649 |
| 6 | 0 | 0 | 0 | 1946 |  | -40 | 0 | 1906 |  |
| 6 | 0 | 11 | 41 |  | 1040 | -40 | 701 |  | 1000 |
| 7 | 0 | 0 | 0 | 1705 |  | -40 | 0 | 1665 |  |
| 7 | 0 | 17 | 21 |  | 375 | -40 | 1041 |  | 335 |

## (10) Water-absorbing content:30.00 g/m^2^; Adhesive content:5.00 g/m^2^; Weathered red-bed soil 5.00%:

| Start: t_1_ (min) | Finish: t_2_ | Duration: t | To start the water head: h1(cm) | End the water head:h_2_ (cm) | $2.3\frac{a}{A}\frac{L}{t}$ | $lg\frac{h_{1}}{h_{2}}$ | Permeability coefficient at water temperature T °C:k_T_ (cm/s) | Water temperature (℃) | Correction coefficient | Permeability coefficient: k_20_ (cm/s) | Average permeability coefficient:k_20_ (cm/s) |
| --- | --- | --- | --- | --- | --- | --- | --- | --- | --- | --- | --- |
| 1 | 2 | 3 | 4 | 5 | 6 | 7 | 8 | 9 | 10 | 11 | 12 |
|  |  | (2)-(1) |  |  | $2.3\frac{a}{A}\frac{L}{(3)}$ | $lg\frac{(4)}{(5)}$ | (6)×(7) |  |  | (8)×(10) | $\frac{\sum(11)}{n}$ |
| 0 | 875 | 875 | 1885 | 695 | 0.0001829 | 0.433 | 0.0000792 | 27 | 0.85 | 0.0000673 | 0.000059 |
| 0 | 740 | 740 | 1944 | 881 | 0.0002162 | 0.3437 | 0.0000743 | 27 | 0.85 | 0.0000632 |  |
| 0 | 1554 | 1554 | 1899 | 385 | 0.000103 | 0.6931 | 0.0000714 | 27 | 0.85 | 0.0000607 |  |
| 0 | 804 | 804 | 1759 | 814 | 0.0001990 | 0.3345 | 0.0000666 | 27 | 0.85 | 0.0000566 |  |
| 0 | 1029 | 1029 | 1795 | 719 | 0.0001555 | 0.3973 | 0.0000618 | 27 | 0.85 | 0.0000525 |  |
| 0 | 1320 | 1320 | 1818 | 550 | 0.0001212 | 0.5192 | 0.0000629 | 27 | 0.85 | 0.0000535 |  |
| 0 | 4641 | 4641 | 2135 | 805 | 0.0000345 | 0.4236 | 0.0000146 | 17 |  |  |  |

**Record of water head:**

| Times | Hour | Minute | Second | Start water head | Ended  water head | correction number | Total seconds | Corrects start water head | Corrects ended water head |
| --- | --- | --- | --- | --- | --- | --- | --- | --- | --- |
| 1 | 0 | 0 | 0 | 1920 |  | -35 | 0 | 1885 |  |
| 1 | 0 | 14 | 35 |  | 730 | -35 | 875 |  | 695 |
| 2 | 0 | 0 | 0 | 1979 |  | -35 | 0 | 1944 |  |
| 2 | 0 | 12 | 20 |  | 916 | -35 | 740 |  | 881 |
| 3 | 0 | 0 | 0 | 1934 |  | -35 | 0 | 1899 |  |
| 3 | 0 | 25 | 54 |  | 420 | -35 | 1554 |  | 385 |
| 4 | 0 | 0 | 0 | 1794 |  | -35 | 0 | 1759 |  |
| 4 | 0 | 13 | 24 |  | 849 | -35 | 804 |  | 814 |
| 5 | 0 | 0 | 0 | 1830 |  | -35 | 0 | 1795 |  |
| 5 | 0 | 17 | 9 |  | 754 | -35 | 1029 |  | 719 |
| 6 | 0 | 0 | 0 | 1853 |  | -35 | 0 | 1818 |  |
| 6 | 0 | 22 | 0 |  | 585 | -35 | 1320 |  | 550 |
| 7 | 0 | 0 | 0 | 1705 |  | 430 | 0 | 2135 |  |
| 7 | 1 | 17 | 21 |  | 375 | 430 | 4641 |  | 805 |

## (11) Water-absorbing content:15.00 g/m^2^; Adhesive content:2.50 g/m^2^; Weathered red-bed soil 0.00%:

| Start: t_1_ (min) | Finish: t_2_ | Duration: t | To start the water head: h1(cm) | End the water head:h_2_ (cm) | $2.3\frac{a}{A}\frac{L}{t}$ | $lg\frac{h_{1}}{h_{2}}$ | Permeability coefficient at water temperature T °C:k_T_ (cm/s) | Water temperature (℃) | Correction coefficient | Permeability coefficient: k_20_ (cm/s) | Average permeability coefficient:k_20_ (cm/s) |
| --- | --- | --- | --- | --- | --- | --- | --- | --- | --- | --- | --- |
| 1 | 2 | 3 | 4 | 5 | 6 | 7 | 8 | 9 | 10 | 11 | 12 |
|  |  | (2)-(1) |  |  |  |  | (6)×(7) |  |  | (8)×(10) | $\frac{\sum(11)}{n}$ |
| 0 | 1035 | 1035 | 1867 | 799 | 0.0001546 | 0.3692 | 0.0000571 | 27 | 0.85 | 0.0000485 | 4E6-05 |
| 0 | 1143 | 1143 | 1756 | 701 | 0.00014 | 0.3988 | 0.0000558 | 27 | 0.85 | 0.0000474 |  |
| 0 | 1263 | 1263 | 1631 | 612 | 0.0001267 | 0.4257 | 0.0000539 | 27 | 0.85 | 0.0000458 |  |
| 0 | 1232 | 1232 | 1695 | 670 | 0.0001299 | 0.4031 | 0.0000524 | 27 | 0.85 | 0.0000445 |  |
| 0 | 1609 | 1609 | 1589 | 475 | 0.0000994 | 0.5244 | 0.0000521 | 27 | 0.85 | 0.0000443 |  |
| 0 | 1189 | 1189 | 1875 | 800 | 0.0001346 | 0.3699 | 0.0000498 | 27 | 0.85 | 0.0000423 |  |
| 0 | 1035 | 1035 | 1867 | 799 | 0.0001546 | 0.3692 | 0.0000571 | 27 | 0.85 | 0.0000485 |  |

**Record of water head:**

| Times | Hour | Minute | Second | Start water head | Ended  water head | correction number | Total seconds | Corrects start water head | Corrects ended water head |
| --- | --- | --- | --- | --- | --- | --- | --- | --- | --- |
| 1 | 0 | 0 | 0 | 1902 |  | -35 | 0 | 1867 |  |
| 1 | 0 | 17 | 15 |  | 834 | -35 | 1035 |  | 799 |
| 2 | 0 | 0 | 0 | 1791 |  | -35 | 0 | 1756 |  |
| 2 | 0 | 19 | 3 |  | 736 | -35 | 1143 |  | 701 |
| 3 | 0 | 0 | 0 | 1666 |  | -35 | 0 | 1631 |  |
| 3 | 0 | 21 | 3 |  | 647 | -35 | 1263 |  | 612 |
| 4 | 0 | 0 | 0 | 1730 |  | -35 | 0 | 1695 |  |
| 4 | 0 | 20 | 32 |  | 705 | -35 | 1232 |  | 670 |
| 5 | 0 | 0 | 0 | 1624 |  | -35 | 0 | 1589 |  |
| 5 | 0 | 26 | 49 |  | 510 | -35 | 1609 |  | 475 |
| 6 | 0 | 0 | 0 | 1910 |  | -35 | 0 | 1875 |  |
| 6 | 0 | 19 | 49 |  | 835 | -35 | 1189 |  | 800 |

## (12) Water-absorbing content:15.00 g/m^2^; Adhesive content:2.50 g/m^2^; Weathered red-bed soil 1.00%:

| Start: t_1_ (min) | Finish: t_2_ | Duration: t | To start the water head: h1(cm) | End the water head:h_2_ (cm) | $2.3\frac{a}{A}\frac{L}{t}$ | $lg\frac{h_{1}}{h_{2}}$ | Permeability coefficient at water temperature T °C:k_T_ (cm/s) | Water temperature (℃) | Correction coefficient | Permeability coefficient: k_20_ (cm/s) | Average permeability coefficient:k_20_ (cm/s) |
| --- | --- | --- | --- | --- | --- | --- | --- | --- | --- | --- | --- |
| 1 | 2 | 3 | 4 | 5 | 6 | 7 | 8 | 9 | 10 | 11 | 12 |
|  |  | (2)-(1) |  |  | $2.3\frac{a}{A}\frac{L}{(3)}$ | $lg\frac{(4)}{(5)}$ | (6)×(7) |  |  | (8)×(10) | $\frac{\sum(11)}{n}$ |
| 0 | 1219 | 1219 | 1699 | 368 | 0.0001313 | 0.6646 | 0.0000873 | 27 | 0.85 | 0.0000742 | 6.73E-05 |
| 0 | 1106 | 1106 | 1837 | 465 | 0.0001447 | 0.5967 | 0.0000863 | 27 | 0.85 | 0.0000734 |  |
| 0 | 1054 | 1054 | 1779 | 518 | 0.0001518 | 0.5358 | 0.0000813 | 27 | 0.85 | 0.0000691 |  |
| 0 | 995 | 995 | 1743 | 584 | 0.0001608 | 0.4742 | 0.0000763 | 27 | 0.85 | 0.0000649 |  |
| 0 | 962 | 962 | 1724 | 625 | 0.0001663 | 0.4407 | 0.0000733 | 27 | 0.85 | 0.0000623 |  |
| 0 | 857 | 857 | 1760 | 739 | 0.0001867 | 0.3769 | 0.0000704 | 27 | 0.85 | 0.0000598 |  |
| 0 | 4695 | 4695 | 2030 | 695 | 0.0000341 | 0.4655 | 0.0000159 | 17 | 1.077 | 0.0000171 |  |

**Record of water head:**

| Times | Hour | Minute | Second | Start water head | Ended  water head | correction number | Total seconds | Corrects start water head | Corrects ended water head |
| --- | --- | --- | --- | --- | --- | --- | --- | --- | --- |
| 1 | 0 | 0 | 0 | 1734 |  | -35 | 0 | 1699 |  |
| 1 | 0 | 20 | 19 |  | 403 | -35 | 1219 |  | 368 |
| 2 | 0 | 0 | 0 | 1872 |  | -35 | 0 | 1837 |  |
| 2 | 0 | 18 | 26 |  | 500 | -35 | 1106 |  | 465 |
| 3 | 0 | 0 | 0 | 1814 |  | -35 | 0 | 1779 |  |
| 3 | 0 | 17 | 34 |  | 553 | -35 | 1054 |  | 518 |
| 4 | 0 | 0 | 0 | 1778 |  | -35 | 0 | 1743 |  |
| 4 | 0 | 16 | 35 |  | 619 | -35 | 995 |  | 584 |
| 5 | 0 | 0 | 0 | 1759 |  | -35 | 0 | 1724 |  |
| 5 | 0 | 16 | 2 |  | 660 | -35 | 962 |  | 625 |
| 6 | 0 | 0 | 0 | 1795 |  | -35 | 0 | 1760 |  |
| 6 | 0 | 14 | 17 |  | 774 | -35 | 857 |  | 739 |
| 7 | 0 | 0 | 0 | 1560 |  | 470 | 0 | 2030 |  |
| 7 | 1 | 18 | 15 |  | 225 | 470 | 4695 |  | 695 |

## (13) Water-absorbing content:15.00 g/m^2^; Adhesive content:2.50 g/m^2^; Weathered red-bed soil 2.50%:

| Start: t_1_ (min) | Finish: t_2_ | Duration: t | To start the water head: h1(cm) | End the water head:h_2_ (cm) | $2.3\frac{a}{A}\frac{L}{t}$ | $lg\frac{h_{1}}{h_{2}}$ | Permeability coefficient at water temperature T °C:k_T_ (cm/s) | Water temperature (℃) | Correction coefficient | Permeability coefficient: k_20_ (cm/s) | Average permeability coefficient:k_20_ (cm/s) |
| --- | --- | --- | --- | --- | --- | --- | --- | --- | --- | --- | --- |
| 1 | 2 | 3 | 4 | 5 | 6 | 7 | 8 | 9 | 10 | 11 | 12 |
|  |  | (2)-(1) |  |  | $2.3\frac{a}{A}\frac{L}{(3)}$ | $lg\frac{(4)}{(5)}$ | (6)×(7) |  |  | (8)×(10) | $\frac{\sum(11)}{n}$ |
| 0 | 1219 | 1219 | 1960 | 470 | 0.0001313 | 0.6201 | 0.0000814 | 27 | 0.85 | 0.0000692 | 0.0000574 |
| 0 | 1051 | 1051 | 1770 | 584 | 0.0001522 | 0.4816 | 0.0000733 | 27 | 0.85 | 0.0000623 |  |
| 0 | 1033 | 1033 | 1820 | 696 | 0.0001549 | 0.4175 | 0.0000647 | 27 | 0.85 | 0.000055 |  |
| 0 | 960 | 960 | 1712 | 717 | 0.0001667 | 0.3784 | 0.0000631 | 27 | 0.85 | 0.0000536 |  |
| 0 | 945 | 945 | 1710 | 740 | 0.0001693 | 0.3638 | 0.0000616 | 27 | 0.85 | 0.0000524 |  |
| 0 | 931 | 931 | 1700 | 752 | 0.0001719 | 0.3542 | 0.0000609 | 27 | 0.85 | 0.0000518 |  |
| 0 | 4641 | 4641 | 1675 | 345 | 0.0000345 | 0.6862 | 0.0000237 | 17 | 1.077 | 0.0000255 |  |

**Record of water head:**

| Times | Hour | Minute | Second | Start water head | Ended  water head | correction number | Total seconds | Corrects start water head | Corrects ended water head |
| --- | --- | --- | --- | --- | --- | --- | --- | --- | --- |
| 1 | 0 | 0 | 0 | 1990 |  | -30 | 0 | 1960 |  |
| 1 | 0 | 20 | 19 |  | 500 | -30 | 1219 |  | 470 |
| 2 | 0 | 0 | 0 | 1800 |  | -30 | 0 | 1770 |  |
| 2 | 0 | 17 | 31 |  | 614 | -30 | 1051 |  | 584 |
| 3 | 0 | 0 | 0 | 1850 |  | -30 | 0 | 1820 |  |
| 3 | 0 | 17 | 13 |  | 726 | -30 | 1033 |  | 696 |
| 4 | 0 | 0 | 0 | 1742 |  | -30 | 0 | 1712 |  |
| 4 | 0 | 16 | 0 |  | 747 | -30 | 960 |  | 717 |
| 5 | 0 | 0 | 0 | 1740 |  | -30 | 0 | 1710 |  |
| 5 | 0 | 15 | 45 |  | 770 | -30 | 945 |  | 740 |
| 6 | 0 | 0 | 0 | 1730 |  | -30 | 0 | 1700 |  |
| 6 | 0 | 15 | 31 |  | 782 | -30 | 931 |  | 752 |
| 7 | 0 | 0 | 0 | 1705 |  | -30 | 0 | 1675 |  |
| 7 | 1 | 17 | 21 |  | 375 | -30 | 4641 |  | 345 |

## (14) Water-absorbing content:15.00 g/m^2^; Adhesive content:2.50 g/m^2^; Weathered red-bed soil 5.00%:

| Start: t_1_ (min) | Finish: t_2_ | Duration: t | To start the water head: h1(cm) | End the water head:h_2_ (cm) | $2.3\frac{a}{A}\frac{L}{t}$ | $lg\frac{h_{1}}{h_{2}}$ | Permeability coefficient at water temperature T °C:k_T_ (cm/s) | Water temperature (℃) | Correction coefficient | Permeability coefficient: k_20_ (cm/s) | Average permeability coefficient:k_20_ (cm/s) |
| --- | --- | --- | --- | --- | --- | --- | --- | --- | --- | --- | --- |
| 1 | 2 | 3 | 4 | 5 | 6 | 7 | 8 | 9 | 10 | 11 | 12 |
|  |  | (2)-(1) |  |  | $2.3\frac{a}{A}\frac{L}{(3)}$ | $lg\frac{(4)}{(5)}$ | (6)×(7) |  |  | (8)×(10) | $\frac{\sum(11)}{n}$ |
| 0 | 1676 | 1676 | 1945 | 710 | 0.0000955 | 0.4378 | 0.0000418 | 27 | 0.85 | 0.0000355 | 0.0000335 |
| 0 | 1557 | 1557 | 1875 | 752 | 0.0001028 | 0.3968 | 0.0000408 | 27 | 0.85 | 0.0000347 |  |
| 0 | 1932 | 1932 | 1809 | 594 | 0.0000828 | 0.4837 | 0.0000401 | 27 | 0.85 | 0.0000341 |  |
| 0 | 1328 | 1328 | 1793 | 858 | 0.0001205 | 0.3201 | 0.0000386 | 27 | 0.85 | 0.0000328 |  |
| 0 | 2222 | 2222 | 1800 | 528 | 0.0000720 | 0.5326 | 0.0000383 | 27 | 0.85 | 0.0000326 |  |
| 0 | 3851 | 3851 | 1790 | 228 | 0.0000415 | 0.8949 | 0.0000371 | 27 | 0.85 | 0.0000315 |  |
| 0 | 4641 | 4641 | 2135 | 805 | 0.0000345 | 0.4236 | 0.0000146 | 17 |  |  |  |

**Record of water head:**

| Times | Hour | Minute | Second | Start water head | Ended  water head | correction number | Total seconds | Corrects start water head | Corrects ended water head |
| --- | --- | --- | --- | --- | --- | --- | --- | --- | --- |
| 1 | 0 | 0 | 0 | 1975 |  | -30 | 0 | 1945 |  |
| 1 | 0 | 27 | 56 |  | 740 | -30 | 1676 |  | 710 |
| 2 | 0 | 0 | 0 | 1905 |  | -30 | 0 | 1875 |  |
| 2 | 0 | 25 | 57 |  | 782 | -30 | 1557 |  | 752 |
| 3 | 0 | 0 | 0 | 1839 |  | -30 | 0 | 1809 |  |
| 3 | 0 | 32 | 12 |  | 624 | -30 | 1932 |  | 594 |
| 4 | 0 | 0 | 0 | 1823 |  | -30 | 0 | 1793 |  |
| 4 | 0 | 22 | 8 |  | 888 | -30 | 1328 |  | 858 |
| 5 | 0 | 0 | 0 | 1830 |  | -30 | 0 | 1800 |  |
| 5 | 0 | 37 | 2 |  | 558 | -30 | 2222 |  | 528 |
| 6 | 0 | 0 | 0 | 1820 |  | -30 | 0 | 1790 |  |
| 6 | 1 | 4 | 11 |  | 258 | -30 | 3851 |  | 228 |
| 7 | 0 | 0 | 0 | 1705 |  | 430 | 0 | 2135 |  |
| 7 | 1 | 17 | 21 |  | 375 | 430 | 4641 |  | 805 |

## (15) Water-absorbing content:15.00 g/m^2^; Adhesive content:2.50 g/m^2^; Weathered red-bed soil 10.00%:

| Start: t_1_ (min) | Finish: t_2_ | Duration: t | To start the water head: h1(cm) | End the water head:h_2_ (cm) | $2.3\frac{a}{A}\frac{L}{t}$ | $lg\frac{h_{1}}{h_{2}}$ | Permeability coefficient at water temperature T °C:k_T_ (cm/s) | Water temperature (℃) | Correction coefficient | Permeability coefficient: k_20_ (cm/s) | Average permeability coefficient:k_20_ (cm/s) |
| --- | --- | --- | --- | --- | --- | --- | --- | --- | --- | --- | --- |
| 1 | 2 | 3 | 4 | 5 | 6 | 7 | 8 | 9 | 10 | 11 | 12 |
|  |  | (2)-(1) |  |  | $2.3\frac{a}{A}\frac{L}{(3)}$ | $lg\frac{(4)}{(5)}$ | (6)×(7) |  |  | (8)×(10) | $\frac{\sum(11)}{n}$ |
| 0 | 1343 | 1343 | 1810 | 781 | 0.0001191 | 0.3655 | 0.0000435 | 27 | 0.85 | 0.000037 | 0.0000333 |
| 0 | 1397 | 1397 | 1751 | 779 | 0.0001145 | 0.3517 | 0.0000403 | 27 | 0.85 | 0.0000343 |  |
| 0 | 1078 | 1078 | 1580 | 872 | 0.0001484 | 0.2581 | 0.0000383 | 27 | 0.85 | 0.0000326 |  |
| 0 | 2195 | 2195 | 1640 | 497 | 0.0000729 | 0.5185 | 0.0000378 | 27 | 0.85 | 0.0000321 |  |
| 0 | 1693 | 1693 | 1575 | 654 | 0.0000945 | 0.3817 | 0.0000361 | 27 | 0.85 | 0.0000307 |  |
| 0 | 2110 | 2110 | 1610 | -30 | 0.0000758 |  |  | 27 | 0.85 |  |  |
| 0 | 0 | 0 | 1675 | -30 |  |  |  | 27 | 0.85 |  |  |

**Record of water head:**

| Times | Hour | Minute | Second | Start water head | Ended  water head | correction number | Total seconds | Corrects start water head | Corrects ended water head |
| --- | --- | --- | --- | --- | --- | --- | --- | --- | --- |
| 1 | 0 | 0 | 0 | 1840 |  | -30 | 0 | 1810 |  |
| 1 | 0 | 22 | 23 |  | 811 | -30 | 1343 |  | 781 |
| 2 | 0 | 0 | 0 | 1781 |  | -30 | 0 | 1751 |  |
| 2 | 0 | 23 | 17 |  | 809 | -30 | 1397 |  | 779 |
| 3 | 0 | 0 | 0 | 1610 |  | -30 | 0 | 1580 |  |
| 3 | 0 | 17 | 58 |  | 902 | -30 | 1078 |  | 872 |
| 4 | 0 | 0 | 0 | 1670 |  | -30 | 0 | 1640 |  |
| 4 | 0 | 36 | 35 |  | 527 | -30 | 2195 |  | 497 |
| 5 | 0 | 0 | 0 | 1605 |  | -30 | 0 | 1575 |  |
| 5 | 0 | 28 | 13 |  | 684 | -30 | 1693 |  | 654 |
| 6 | 0 | 0 | 0 | 1640 |  | -30 | 0 | 1610 |  |
| 6 | 0 | 35 | 10 |  | 0 | -30 | 2110 |  | -30 |
| 7 | 0 | 0 | 0 | 1705 |  | -30 | 0 | 1675 |  |
| 7 | 0 | 0 | 0 |  | 0 | -30 | 0 |  | -30 |

## (16) Water-absorbing content:30.00 g/m^2^; Adhesive content:2.50 g/m^2^; Weathered red-bed soil 0.00%:

| Start: t_1_ (min) | Finish: t_2_ | Duration: t | To start the water head: h1(cm) | End the water head:h_2_ (cm) | $2.3\frac{a}{A}\frac{L}{t}$ | $lg\frac{h_{1}}{h_{2}}$ | Permeability coefficient at water temperature T °C:k_T_ (cm/s) | Water temperature (℃) | Correction coefficient | Permeability coefficient: k_20_ (cm/s) | Average permeability coefficient:k_20_ (cm/s) |
| --- | --- | --- | --- | --- | --- | --- | --- | --- | --- | --- | --- |
| 1 | 2 | 3 | 4 | 5 | 6 | 7 | 8 | 9 | 10 | 11 | 12 |
|  |  | (2)-(1) |  |  | $2.3\frac{a}{A}\frac{L}{(3)}$ | $lg\frac{(4)}{(5)}$ | (6)×(7) |  |  | (8)×(10) | $\frac{\sum(11)}{n}$ |
| 0 | 384 | 384 | 1566 | 616 | 0.0004167 | 0.4048 | 0.0001687 | 26 | 0.87 | 0.0001468 | 0.00012 |
| 0 | 551 | 551 | 1824 | 540 | 0.0002904 | 0.5286 | 0.0001535 | 26 | 0.87 | 0.0001335 |  |
| 0 | 468 | 468 | 1622 | 647 | 0.0003419 | 0.3991 | 0.0001365 | 26 | 0.87 | 0.0001188 |  |
| 0 | 664 | 664 | 1761 | 483 | 0.0002410 | 0.5623 | 0.0001355 | 26 | 0.87 | 0.0001179 |  |
| 0 | 798 | 798 | 1735 | 415 | 0.0002005 | 0.6213 | 0.0001246 | 26 | 0.87 | 0.0001084 |  |
| 0 | 628 | 628 | 1801 | 551 | 0.0002548 | 0.5144 | 0.0001311 | 26 | 0.87 | 0.0001141 |  |

**Record of water head:**

| Times | Hour | Minute | Second | Start water head | Ended  water head | correction number | Total seconds | Corrects start water head | Corrects ended water head |
| --- | --- | --- | --- | --- | --- | --- | --- | --- | --- |
| 1 | 0 | 0 | 0 | 1601 |  | -35 | 0 | 1566 |  |
| 1 | 0 | 6 | 24 |  | 651 | -35 | 384 |  | 616 |
| 2 | 0 | 0 | 0 | 1859 |  | -35 | 0 | 1824 |  |
| 2 | 0 | 9 | 11 |  | 575 | -35 | 551 |  | 540 |
| 3 | 0 | 0 | 0 | 1657 |  | -35 | 0 | 1622 |  |
| 3 | 0 | 7 | 48 |  | 682 | -35 | 468 |  | 647 |
| 4 | 0 | 0 | 0 | 1796 |  | -35 | 0 | 1761 |  |
| 4 | 0 | 11 | 4 |  | 518 | -35 | 664 |  | 483 |
| 5 | 0 | 0 | 0 | 1770 |  | -35 | 0 | 1735 |  |
| 5 | 0 | 13 | 18 |  | 450 | -35 | 798 |  | 415 |
| 6 | 0 | 0 | 0 | 1836 |  | -35 | 0 | 1801 |  |
| 6 | 0 | 10 | 28 |  | 586 | -35 | 628 |  | 551 |
| 7 | 0 | 0 | 0 | 1797 |  | -35 | 0 | 1762 |  |
| 7 | 0 | 8 | 24 |  | 740 | -35 | 504 |  | 705 |

## (17) Water-absorbing content:30.00 g/m^2^; Adhesive content:2.50 g/m^2^; Weathered red-bed soil 1.00%:

| Start: t_1_ (min) | Finish: t_2_ | Duration: t | To start the water head: h1(cm) | End the water head:h_2_ (cm) | $2.3\frac{a}{A}\frac{L}{t}$ | $lg\frac{h_{1}}{h_{2}}$ | Permeability coefficient at water temperature T °C:k_T_ (cm/s) | Water temperature (℃) | Correction coefficient | Permeability coefficient: k_20_ (cm/s) | Average permeability coefficient:k_20_ (cm/s) |
| --- | --- | --- | --- | --- | --- | --- | --- | --- | --- | --- | --- |
| 1 | 2 | 3 | 4 | 5 | 6 | 7 | 8 | 9 | 10 | 11 | 12 |
|  |  | (2)-(1) |  |  | $2.3\frac{a}{A}\frac{L}{(3)}$ | $lg\frac{(4)}{(5)}$ | (6)×(7) |  |  | (8)×(10) | $\frac{\sum(11)}{n}$ |
| 0 | 2183 | 2183 | 1786 | 699 | 0.0000733 | 0.4082 | 0.0000299 | 30 | 0.798 | 0.0000239 | 2.27E-05 |
| 0 | 2287 | 2287 | 1701 | 683 | 0.00007 | 0.3963 | 0.0000277 | 30 | 0.798 | 0.0000221 |  |
| 0 | 3223 | 3223 | 1721 | 518 | 0.0000496 | 0.5215 | 0.0000259 | 27 | 0.85 | 0.000022 |  |
| 0 | 2064 | 2064 | 1714 | 818 | 0.0000775 | 0.3222 | 0.000025 | 27 | 0.85 | 0.0000213 |  |
| 0 | 882 | 882 | 1950 | 1330 | 0.0001814 | 0.1662 | 0.0000301 | 27 | 0.85 | 0.0000256 |  |
| 0 | 1805 | 1805 | 1950 | 1023 | 0.0000886 | 0.2802 | 0.0000248 | 27 | 0.85 | 0.0000211 |  |
| 0 | 2496 | 2496 | 1755 | 630 | 0.0000641 | 0.4449 | 0.0000285 | 27 | 0.85 | 0.0000242 |  |

**Record of water head:**

| Times | Hour | Minute | Second | Start water head | Ended  water head | correction number | Total seconds | Corrects start water head | Corrects ended water head |
| --- | --- | --- | --- | --- | --- | --- | --- | --- | --- |
| 1 | 0 | 0 | 0 | 1816 |  | -30 | 0 | 1786 |  |
| 1 | 0 | 36 | 23 |  | 729 | -30 | 2183 |  | 699 |
| 2 | 0 | 0 | 0 | 1731 |  | -30 | 0 | 1701 |  |
| 2 | 0 | 38 | 7 |  | 713 | -30 | 2287 |  | 683 |
| 3 | 0 | 0 | 0 | 1751 |  | -30 | 0 | 1721 |  |
| 3 | 0 | 53 | 43 |  | 548 | -30 | 3223 |  | 518 |
| 4 | 0 | 0 | 0 | 1744 |  | -30 | 0 | 1714 |  |
| 4 | 0 | 34 | 24 |  | 848 | -30 | 2064 |  | 818 |
| 5 | 0 | 0 | 0 | 1980 |  | -30 | 0 | 1950 |  |
| 5 | 0 | 14 | 42 |  | 1360 | -30 | 882 |  | 1330 |
| 6 | 0 | 0 | 0 | 1980 |  | -30 | 0 | 1950 |  |
| 6 | 0 | 30 | 5 |  | 1053 | -30 | 1805 |  | 1023 |
| 7 | 0 | 0 | 0 | 1785 |  | -30 | 0 | 1755 |  |
| 7 | 0 | 41 | 36 |  | 660 | -30 | 2496 |  | 630 |

## (18) Water-absorbing content:30.00 g/m^2^; Adhesive content:2.50 g/m^2^; Weathered red-bed soil 2.50%:

| Start: t_1_ (min) | Finish: t_2_ | Duration: t | To start the water head: h1(cm) | End the water head:h_2_ (cm) | $2.3\frac{a}{A}\frac{L}{t}$ | $lg\frac{h_{1}}{h_{2}}$ | Permeability coefficient at water temperature T °C:k_T_ (cm/s) | Water temperature (℃) | Correction coefficient | Permeability coefficient: k_20_ (cm/s) | Average permeability coefficient:k_20_ (cm/s) |
| --- | --- | --- | --- | --- | --- | --- | --- | --- | --- | --- | --- |
| 1 | 2 | 3 | 4 | 5 | 6 | 7 | 8 | 9 | 10 | 11 | 12 |
|  |  | (2)-(1) |  |  | $2.3\frac{a}{A}\frac{L}{(3)}$ | $lg\frac{(4)}{(5)}$ | (6)×(7) |  |  | (8)×(10) | $\frac{\sum(11)}{n}$ |
| 0 | 1440 | 1440 | 1610 | 483 | 0.0001111 | 0.5224 | 0.000058 | 29 | 0.815 | 0.0000473 | 0.0000433 |
| 0 | 878 | 878 | 1914 | 978 | 0.0001822 | 0.2916 | 0.0000531 | 29 | 0.815 | 0.0000433 |  |
| 0 | 1026 | 1026 | 1703 | 798 | 0.0001559 | 0.3292 | 0.0000513 | 27 | 0.85 | 0.0000436 |  |
| 0 | 2409 | 2409 | 1703 | 295 | 0.0000664 | 0.7612 | 0.0000505 | 27 | 0.85 | 0.0000429 |  |
| 0 | 1181 | 1181 | 1940 | 854 | 0.0001355 | 0.3563 | 0.0000483 | 27 | 0.85 | 0.0000411 |  |
| 0 | 2584 | 2584 | 1940 | 316 | 0.0000619 | 0.7881 | 0.0000488 | 27 | 0.85 | 0.0000415 |  |
| 0 | 4641 | 4641 | 1670 | 340 | 0.0000345 | 0.6912 | 0.0000238 | 17 | 1.077 | 0.0000256 |  |

**Record of water head:**

| Times | Hour | Minute | Second | Start water head | Ended  water head | correction number | Total seconds | Corrects start water head | Corrects ended water head |
| --- | --- | --- | --- | --- | --- | --- | --- | --- | --- |
| 1 | 0 | 0 | 0 | 1645 |  | -35 | 0 | 1610 |  |
| 1 | 0 | 24 | 0 |  | 518 | -35 | 1440 |  | 483 |
| 2 | 0 | 0 | 0 | 1949 |  | -35 | 0 | 1914 |  |
| 2 | 0 | 14 | 38 |  | 1013 | -35 | 878 |  | 978 |
| 3 | 0 | 0 | 0 | 1738 |  | -35 | 0 | 1703 |  |
| 3 | 0 | 17 | 6 |  | 833 | -35 | 1026 |  | 798 |
| 4 | 0 | 0 | 0 | 1738 |  | -35 | 0 | 1703 |  |
| 4 | 0 | 40 | 9 |  | 330 | -35 | 2409 |  | 295 |
| 5 | 0 | 0 | 0 | 1975 |  | -35 | 0 | 1940 |  |
| 5 | 0 | 19 | 41 |  | 889 | -35 | 1181 |  | 854 |
| 6 | 0 | 0 | 0 | 1975 |  | -35 | 0 | 1940 |  |
| 6 | 0 | 43 | 4 |  | 351 | -35 | 2584 |  | 316 |
| 7 | 0 | 0 | 0 | 1705 |  | -35 | 0 | 1670 |  |
| 7 | 1 | 17 | 21 |  | 375 | -35 | 4641 |  | 340 |

## (19) Water-absorbing content:30.00 g/m^2^; Adhesive content:2.50 g/m^2^; Weathered red-bed soil 0.00%:

| Start: t_1_ (min) | Finish: t_2_ | Duration: t | To start the water head: h1(cm) | End the water head:h_2_ (cm) | $2.3\frac{a}{A}\frac{L}{t}$ | $lg\frac{h_{1}}{h_{2}}$ | Permeability coefficient at water temperature T °C:k_T_ (cm/s) | Water temperature (℃) | Correction coefficient | Permeability coefficient: k_20_ (cm/s) | Average permeability coefficient:k_20_ (cm/s) |
| --- | --- | --- | --- | --- | --- | --- | --- | --- | --- | --- | --- |
| 1 | 2 | 3 | 4 | 5 | 6 | 7 | 8 | 9 | 10 | 11 | 12 |
|  |  | (2)-(1) |  |  | $2.3\frac{a}{A}\frac{L}{(3)}$ | $lg\frac{(4)}{(5)}$ | (6)×(7) |  |  | (8)×(10) | $\frac{\sum(11)}{n}$ |
|  |  | (2)-(1) |  |  |  |  | (6)×(7) |  |  | (8)×(10) | 0.0000426 |
| 0 | 1219 | 1219 | 1833 | 626 | 0.0001313 | 0.4669 | 0.0000613 | 27 | 0.85 | 0.0000521 |  |
| 0 | 1125 | 1125 | 1834 | 773 | 0.0001422 | 0.3752 | 0.0000534 | 27 | 0.85 | 0.0000454 |  |
| 0 | 1070 | 1070 | 1781 | 828 | 0.0001495 | 0.3326 | 0.0000497 | 27 | 0.85 | 0.0000422 |  |
| 0 | 1008 | 1008 | 1805 | 908 | 0.0001587 | 0.2989 | 0.0000474 | 27 | 0.85 | 0.0000403 |  |
| 0 | 966 | 966 | 1686 | 897 | 0.0001656 | 0.2741 | 0.0000454 | 27 | 0.85 | 0.0000386 |  |
| 0 | 1423 | 1423 | 1776 | 733 | 0.0001124 | 0.3843 | 0.0000432 | 27 | 0.85 | 0.0000367 |  |

**Record of water head:**

| Times | Hour | Minute | Second | Start water head | Ended  water head | correction number | Total seconds | Corrects start water head | Corrects ended water head |
| --- | --- | --- | --- | --- | --- | --- | --- | --- | --- |
| 1 | 0 | 0 | 0 | 1873 |  | -40 | 0 | 1833 |  |
| 1 | 0 | 20 | 19 |  | 666 | -40 | 1219 |  | 626 |
| 2 | 0 | 0 | 0 | 1874 |  | -40 | 0 | 1834 |  |
| 2 | 0 | 18 | 45 |  | 813 | -40 | 1125 |  | 773 |
| 3 | 0 | 0 | 0 | 1821 |  | -40 | 0 | 1781 |  |
| 3 | 0 | 17 | 50 |  | 868 | -40 | 1070 |  | 828 |
| 4 | 0 | 0 | 0 | 1845 |  | -40 | 0 | 1805 |  |
| 4 | 0 | 16 | 48 |  | 948 | -40 | 1008 |  | 908 |
| 5 | 0 | 0 | 0 | 1726 |  | -40 | 0 | 1686 |  |
| 5 | 0 | 16 | 6 |  | 937 | -40 | 966 |  | 897 |
| 6 | 0 | 0 | 0 | 1816 |  | -40 | 0 | 1776 |  |
| 6 | 0 | 23 | 43 |  | 773 | -40 | 1423 |  | 733 |
| 7 | 0 | 0 | 0 | 1705 |  | 430 | 0 | 2135 |  |
| 7 | 1 | 17 | 21 |  | 375 | 430 | 4641 |  | 805 |

## (20) Water-absorbing content:30.00 g/m^2^; Adhesive content:2.50 g/m^2^; Weathered red-bed soil 5.00%:

| Start: t_1_ (min) | Finish: t_2_ | Duration: t | To start the water head: h1(cm) | End the water head:h_2_ (cm) | $2.3\frac{a}{A}\frac{L}{t}$ | $lg\frac{h_{1}}{h_{2}}$ | Permeability coefficient at water temperature T °C:k_T_ (cm/s) | Water temperature (℃) | Correction coefficient | Permeability coefficient: k_20_ (cm/s) | Average permeability coefficient:k_20_ (cm/s) |
| --- | --- | --- | --- | --- | --- | --- | --- | --- | --- | --- | --- |
| 1 | 2 | 3 | 4 | 5 | 6 | 7 | 8 | 9 | 10 | 11 | 12 |
|  |  | (2)-(1) |  |  | $2.3\frac{a}{A}\frac{L}{(3)}$ | $lg\frac{(4)}{(5)}$ | (6)×(7) |  |  | (8)×(10) | $\frac{\sum(11)}{n}$ |
| 0 | 2165 | 2165 | 2206 | 816 | 0.0000739 | 0.4314 | 0.0000319 | 30 | 0.798 | 0.0000255 | 0.0000252 |
| 0 | 2300 | 2300 | 2178 | 811 | 0.0000696 | 0.429 | 0.0000299 | 30 | 0.798 | 0.0000239 |  |
| 0 | 3222 | 3222 | 2150 | 667 | 0.0000497 | 0.5083 | 0.0000253 | 27 | 0.85 | 0.0000215 |  |
| 0 | 2064 | 2064 | 2380 | 964 | 0.0000775 | 0.3927 | 0.0000304 | 27 | 0.85 | 0.0000258 |  |
| 0 | 1033 | 1033 | 2285 | 1390 | 0.0001549 | 0.2159 | 0.0000334 | 27 | 0.85 | 0.0000284 |  |
| 0 | 1809 | 1809 | 2285 | 1028 | 0.0000884 | 0.3469 | 0.0000307 | 27 | 0.85 | 0.0000261 |  |
| 0 | 0 | 0 | 2135 | 805 |  | 0.4236 |  | 17 | 1.077 |  |  |

**Record of water head:**

| Times | Hour | Minute | Second | Start water head | Ended  water head | correction number | Total seconds | Corrects start water head | Corrects ended water head |
| --- | --- | --- | --- | --- | --- | --- | --- | --- | --- |
| 1 | 0 | 0 | 0 | 1776 |  | 430 | 0 | 2206 |  |
| 1 | 0 | 36 | 5 |  | 386 | 430 | 2165 |  | 816 |
| 2 | 0 | 0 | 0 | 1748 |  | 430 | 0 | 2178 |  |
| 2 | 0 | 38 | 20 |  | 381 | 430 | 2300 |  | 811 |
| 3 | 0 | 0 | 0 | 1720 |  | 430 | 0 | 2150 |  |
| 3 | 0 | 53 | 42 |  | 237 | 430 | 3222 |  | 667 |
| 4 | 0 | 0 | 0 | 1950 |  | 430 | 0 | 2380 |  |
| 4 | 0 | 34 | 24 |  | 534 | 430 | 2064 |  | 964 |
| 5 | 0 | 0 | 0 | 1855 |  | 430 | 0 | 2285 |  |
| 5 | 0 | 17 | 13 |  | 960 | 430 | 1033 |  | 1390 |
| 6 | 0 | 0 | 0 | 1855 |  | 430 | 0 | 2285 |  |
| 6 | 0 | 30 | 9 |  | 598 | 430 | 1809 |  | 1028 |
| 7 | 0 | 0 | 0 | 1705 |  | 430 | 0 | 2135 |  |
| 7 | 0 | 0 | 0 |  | 375 | 430 | 0 |  | 805 |

## (21) Water-absorbing content:15.00 g/m^2^; Adhesive content:5.00 g/m^2^; Weathered red-bed soil 0.00%:

| Start: t_1_ (min) | Finish: t_2_ | Duration: t | To start the water head: h1(cm) | End the water head:h_2_ (cm) | $2.3\frac{a}{A}\frac{L}{t}$ | $lg\frac{h_{1}}{h_{2}}$ | Permeability coefficient at water temperature T °C:k_T_ (cm/s) | Water temperature (℃) | Correction coefficient | Permeability coefficient: k_20_ (cm/s) | Average permeability coefficient:k_20_ (cm/s) |
| --- | --- | --- | --- | --- | --- | --- | --- | --- | --- | --- | --- |
| 1 | 2 | 3 | 4 | 5 | 6 | 7 | 8 | 9 | 10 | 11 | 12 |
|  |  | (2)-(1) |  |  |  |  | (6)×(7) |  |  | (8)×(10) | $\frac{\sum(11)}{n}$ |
| 0 | 973 | 973 | 1766 | 563 | 0.0001644 | 0.4969 | 0.0000817 | 26 | 0.87 | 0.0000711 | 5.6E-05 |
| 0 | 1101 | 1101 | 1707 | 586 | 0.0001453 | 0.4643 | 0.0000675 | 26 | 0.87 | 0.0000587 |  |
| 0 | 1483 | 1483 | 1751 | 460 | 0.0001079 | 0.5805 | 0.0000626 | 26 | 0.87 | 0.0000545 |  |
| 0 | 1507 | 1507 | 1759 | 486 | 0.0001062 | 0.5587 | 0.0000593 | 26 | 0.87 | 0.0000516 |  |
| 0 | 1492 | 1492 | 1728 | 505 | 0.0001072 | 0.5343 | 0.0000573 | 26 | 0.87 | 0.0000499 |  |
| 0 | 1337 | 1337 | 1713 | 594 | 0.0001197 | 0.46 | 0.0000551 | 26 | 0.87 | 0.0000479 |  |
| 0 | 5131 | 5131 | 1685 | 320 | 0.0000312 | 0.7214 | 0.0000225 | 26 | 0.87 | 0.0000196 |  |

**Record of water head:**

| Times | Hour | Minute | Second | Start water head | Ended  water head | correction number | Total seconds | Corrects start water head | Corrects ended water head |
| --- | --- | --- | --- | --- | --- | --- | --- | --- | --- |
| 1 | 0 | 0 | 0 | 1796 |  | -30 | 0 | 1766 |  |
| 1 | 0 | 16 | 13 |  | 593 | -30 | 973 |  | 563 |
| 2 | 0 | 0 | 0 | 1737 |  | -30 | 0 | 1707 |  |
| 2 | 0 | 18 | 21 |  | 616 | -30 | 1101 |  | 586 |
| 3 | 0 | 0 | 0 | 1781 |  | -30 | 0 | 1751 |  |
| 3 | 0 | 24 | 43 |  | 490 | -30 | 1483 |  | 460 |
| 4 | 0 | 0 | 0 | 1789 |  | -30 | 0 | 1759 |  |
| 4 | 0 | 25 | 7 |  | 516 | -30 | 1507 |  | 486 |
| 5 | 0 | 0 | 0 | 1758 |  | -30 | 0 | 1728 |  |
| 5 | 0 | 24 | 52 |  | 535 | -30 | 1492 |  | 505 |
| 6 | 0 | 0 | 0 | 1743 |  | -30 | 0 | 1713 |  |
| 6 | 0 | 22 | 17 |  | 624 | -30 | 1337 |  | 594 |
| 7 | 0 | 0 | 0 | 1715 |  | -30 | 0 | 1685 |  |
| 7 | 1 | 25 | 31 |  | 350 | -30 | 5131 |  | 320 |

## (22) Water-absorbing content:15.00 g/m^2^; Adhesive content:5.00 g/m^2^; Weathered red-bed soil 1.00%:

| Start: t_1_ (min) | Finish: t_2_ | Duration: t | To start the water head: h1(cm) | End the water head:h_2_ (cm) | $2.3\frac{a}{A}\frac{L}{t}$ | $lg\frac{h_{1}}{h_{2}}$ | Permeability coefficient at water temperature T °C:k_T_ (cm/s) | Water temperature (℃) | Correction coefficient | Permeability coefficient: k_20_ (cm/s) | Average permeability coefficient:k_20_ (cm/s) |
| --- | --- | --- | --- | --- | --- | --- | --- | --- | --- | --- | --- |
| 1 | 2 | 3 | 4 | 5 | 6 | 7 | 8 | 9 | 10 | 11 | 12 |
|  |  | (2)-(1) |  |  | $2.3\frac{a}{A}\frac{L}{(3)}$ | $lg\frac{(4)}{(5)}$ | (6)×(7) |  |  | (8)×(10) | $\frac{\sum(11)}{n}$ |
| 0 | 1426 | 1426 | 1732 | 501 | 0.0001122 | 0.5391 | 0.0000605 | 29 | 0.815 | 0.0000493 | 4.49E-05 |
| 0 | 875 | 875 | 1911 | 952 | 0.0001829 | 0.3026 | 0.0000553 | 29 | 0.815 | 0.0000451 |  |
| 0 | 989 | 989 | 1648 | 770 | 0.0001618 | 0.3305 | 0.0000535 | 27 | 0.85 | 0.0000455 |  |
| 0 | 2398 | 2398 | 1648 | 272 | 0.0000667 | 0.7825 | 0.0000522 | 27 | 0.85 | 0.0000444 |  |
| 0 | 1212 | 1212 | 1960 | 810 | 0.0001320 | 0.3838 | 0.0000507 | 27 | 0.85 | 0.0000431 |  |
| 0 | 2574 | 2574 | 1960 | 311 | 0.0000622 | 0.7995 | 0.0000497 | 27 | 0.85 | 0.0000422 |  |
| 0 | 4695 | 4695 | 1520 | 185 | 0.0000341 | 0.9147 | 0.0000312 | 27 | 0.85 | 0.0000265 |  |

**Record of water head:**

| Times | Hour | Minute | Second | Start water head | Ended  water head | correction number | Total seconds | Corrects start water head | Corrects ended water head |
| --- | --- | --- | --- | --- | --- | --- | --- | --- | --- |
| 1 | 0 | 0 | 0 | 1772 | 541 | -40 | 0 | 1732 |  |
| 1 | 0 | 23 | 46 |  | 541 | -40 | 1426 |  | 501 |
| 2 | 0 | 0 | 0 | 1951 |  | -40 | 0 | 1911 |  |
| 2 | 0 | 14 | 35 |  | 992 | -40 | 875 |  | 952 |
| 3 | 0 | 0 | 0 | 1688 |  | -40 | 0 | 1648 |  |
| 3 | 0 | 16 | 29 |  | 810 | -40 | 989 |  | 770 |
| 4 | 0 | 0 | 0 | 1688 |  | -40 | 0 | 1648 |  |
| 4 | 0 | 39 | 58 |  | 312 | -40 | 2398 |  | 272 |
| 5 | 0 | 0 | 0 | 2000 |  | -40 | 0 | 1960 |  |
| 5 | 0 | 20 | 12 |  | 850 | -40 | 1212 |  | 810 |
| 6 | 0 | 0 | 0 | 2000 |  | -40 | 0 | 1960 |  |
| 6 | 0 | 42 | 54 |  | 351 | -40 | 2574 |  | 311 |
| 7 | 0 | 0 | 0 | 1560 |  | -40 | 0 | 1520 |  |
| 7 | 1 | 18 | 15 |  | 225 | -40 | 4695 |  | 185 |

## (23) Water-absorbing content:15.00 g/m^2^; Adhesive content:5.00 g/m^2^; Weathered red-bed soil 2.50%:

| Start: t_1_ (min) | Finish: t_2_ | Duration: t | To start the water head: h1(cm) | End the water head:h_2_ (cm) | $2.3\frac{a}{A}\frac{L}{t}$ | $lg\frac{h_{1}}{h_{2}}$ | Permeability coefficient at water temperature T °C:k_T_ (cm/s) | Water temperature (℃) | Correction coefficient | Permeability coefficient: k_20_ (cm/s) | Average permeability coefficient:k_20_ (cm/s) |
| --- | --- | --- | --- | --- | --- | --- | --- | --- | --- | --- | --- |
| 1 | 2 | 3 | 4 | 5 | 6 | 7 | 8 | 9 | 10 | 11 | 12 |
|  |  | (2)-(1) |  |  | $2.3\frac{a}{A}\frac{L}{(3)}$ | $lg\frac{(4)}{(5)}$ | (6)×(7) |  |  | (8)×(10) | $\frac{\sum(11)}{n}$ |
| 0 | 1352 | 1352 | 1817 | 354 | 0.0001183 | 0.7101 | 0.000084 | 27 | 0.85 | 0.0000714 | 0.0000605 |
| 0 | 1401 | 1401 | 1596 | 351 | 0.0001142 | 0.6577 | 0.0000751 | 27 | 0.85 | 0.0000638 |  |
| 0 | 1078 | 1078 | 1535 | 517 | 0.0001484 | 0.4726 | 0.0000701 | 27 | 0.85 | 0.0000596 |  |
| 0 | 2195 | 2195 | 1671 | 217 | 0.0000729 | 0.8865 | 0.0000646 | 27 | 0.85 | 0.0000549 |  |
| 0 | 1692 | 1692 | 1655 | 365 | 0.0000946 | 0.6565 | 0.0000621 | 27 | 0.85 | 0.0000528 |  |
| 0 | 2110 | 2110 | 1605 | 755 | 0.0000758 | 0.3275 | 0.0000248 | 27 | 0.85 | 0.0000211 |  |
| 0 | 1041 | 1041 | 1670 | 340 | 0.0001537 | 0.6912 | 0.0001062 | 27 | 0.85 | 0.0000903 |  |

**Record of water head:**

| Times | Hour | Minute | Second | Start water head | Ended  water head | correction number | Total seconds | Corrects start water head | Corrects ended water head |
| --- | --- | --- | --- | --- | --- | --- | --- | --- | --- |
| 1 | 0 | 0 | 0 | 1852 |  | -35 | 0 | 1817 |  |
| 1 | 0 | 22 | 32 |  | 389 | -35 | 1352 |  | 354 |
| 2 | 0 | 0 | 0 | 1631 |  | -35 | 0 | 1596 |  |
| 2 | 0 | 23 | 21 |  | 386 | -35 | 1401 |  | 351 |
| 3 | 0 | 0 | 0 | 1570 |  | -35 | 0 | 1535 |  |
| 3 | 0 | 17 | 58 |  | 552 | -35 | 1078 |  | 517 |
| 4 | 0 | 0 | 0 | 1706 |  | -35 | 0 | 1671 |  |
| 4 | 0 | 36 | 35 |  | 252 | -35 | 2195 |  | 217 |
| 5 | 0 | 0 | 0 | 1690 |  | -35 | 0 | 1655 |  |
| 5 | 0 | 28 | 12 |  | 400 | -35 | 1692 |  | 365 |
| 6 | 0 | 0 | 0 | 1640 |  | -35 | 0 | 1605 |  |
| 6 | 0 | 35 | 10 |  | 790 | -35 | 2110 |  | 755 |
| 7 | 0 | 0 | 0 | 1705 |  | -35 | 0 | 1670 |  |
| 7 | 0 | 17 | 21 |  | 375 | -35 | 1041 |  | 340 |

## (24) Water-absorbing content:15.00 g/m^2^; Adhesive content:5.00 g/m^2^; Weathered red-bed soil 5.00%:

| Start: t_1_ (min) | Finish: t_2_ | Duration: t | To start the water head: h1(cm) | End the water head:h_2_ (cm) | $2.3\frac{a}{A}\frac{L}{t}$ | $lg\frac{h_{1}}{h_{2}}$ | Permeability coefficient at water temperature T °C:k_T_ (cm/s) | Water temperature (℃) | Correction coefficient | Permeability coefficient: k_20_ (cm/s) | Average permeability coefficient:k_20_ (cm/s) |
| --- | --- | --- | --- | --- | --- | --- | --- | --- | --- | --- | --- |
| 1 | 2 | 3 | 4 | 5 | 6 | 7 | 8 | 9 | 10 | 11 | 12 |
|  |  | (2)-(1) |  |  | $2.3\frac{a}{A}\frac{L}{(3)}$ | $lg\frac{(4)}{(5)}$ | (6)×(7) |  |  | (8)×(10) | $\frac{\sum(11)}{n}$ |
| 0 | 1360 | 1360 | 1811 | 672 | 0.0001176 | 0.4298 | 0.0000505 | 27 | 0.85 | 0.0000429 | 0.0000408 |
| 0 | 1407 | 1407 | 1611 | 592 | 0.0001137 | 0.4348 | 0.0000494 | 27 | 0.85 | 0.000042 |  |
| 0 | 1078 | 1078 | 1605 | 760 | 0.0001484 | 0.3247 | 0.0000482 | 27 | 0.85 | 0.000041 |  |
| 0 | 2194 | 2194 | 1724 | 400 | 0.0000729 | 0.6345 | 0.0000463 | 27 | 0.85 | 0.0000394 |  |
| 0 | 1692 | 1692 | 1679 | 556 | 0.0000946 | 0.48 | 0.0000454 | 27 | 0.85 | 0.0000386 |  |
| 0 | 0 | 0 | 1600 | 750 |  | 0.3291 |  | 32 |  |  |  |
| 0 | 0 | 0 | -40 | 335 |  |  |  | 33 |  |  |  |

**Record of water head:**

| Times | Hour | Minute | Second | Start water head | Ended  water head | correction number | Total seconds | Corrects start water head | Corrects ended water head |
| --- | --- | --- | --- | --- | --- | --- | --- | --- | --- |
| 1 | 0 | 0 | 0 | 1851 |  | -40 | 0 | 1811 |  |
| 1 | 0 | 22 | 40 |  | 712 | -40 | 1360 |  | 672 |
| 2 | 0 | 0 | 0 | 1651 |  | -40 | 0 | 1611 |  |
| 2 | 0 | 23 | 27 |  | 632 | -40 | 1407 |  | 592 |
| 3 | 0 | 0 | 0 | 1645 |  | -40 | 0 | 1605 |  |
| 3 | 0 | 17 | 58 |  | 800 | -40 | 1078 |  | 760 |
| 4 | 0 | 0 | 0 | 1764 |  | -40 | 0 | 1724 |  |
| 4 | 0 | 36 | 34 |  | 440 | -40 | 2194 |  | 400 |
| 5 | 0 | 0 | 0 | 1719 |  | -40 | 0 | 1679 |  |
| 5 | 0 | 28 | 12 |  | 596 | -40 | 1692 |  | 556 |
| 6 | 0 | 0 | 0 | 1640 |  | -40 | 0 | 1600 |  |
| 6 | 0 | 0 | 0 |  | 790 | -40 | 0 |  | 750 |
| 7 | 0 | 0 | 0 | 0 |  | -40 | 0 | -40 |  |
| 7 | 0 | 0 | 0 |  | 375 | -40 | 0 |  | 335 |

## (25) Water-absorbing content:15.00 g/m^2^; Adhesive content:5.00 g/m^2^; Weathered red-bed soil 10.00%:

| Start: t_1_ (min) | Finish: t_2_ | Duration: t | To start the water head: h1(cm) | End the water head:h_2_ (cm) | $2.3\frac{a}{A}\frac{L}{t}$ | $lg\frac{h_{1}}{h_{2}}$ | Permeability coefficient at water temperature T °C:k_T_ (cm/s) | Water temperature (℃) | Correction coefficient | Permeability coefficient: k_20_ (cm/s) | Average permeability coefficient:k_20_ (cm/s) |
| --- | --- | --- | --- | --- | --- | --- | --- | --- | --- | --- | --- |
| 1 | 2 | 3 | 4 | 5 | 6 | 7 | 8 | 9 | 10 | 11 | 12 |
|  |  | (2)-(1) |  |  | $2.3\frac{a}{A}\frac{L}{(3)}$ | $lg\frac{(4)}{(5)}$ | (6)×(7) |  |  | (8)×(10) | $\frac{\sum(11)}{n}$ |
| 0 | 2176 | 2176 | 1555 | 890 | 0.0000735 | 0.243 | 0.0000179 | 30 | 0.798 | 0.0000143 | 0.0000128 |
| 0 | 2293 | 2293 | 1732 | 1018 | 0.0000698 | 0.2308 | 0.0000161 | 30 | 0.798 | 0.0000128 |  |
| 0 | 3222 | 3222 | 1804 | 908 | 0.0000497 | 0.2982 | 0.0000148 | 27 | 0.85 | 0.0000126 |  |
| 0 | 2064 | 2064 | 1715 | 1119 | 0.0000775 | 0.1847 | 0.0000143 | 27 | 0.85 | 0.0000122 |  |
| 0 | 1080 | 1080 | 1933 | 1535 | 0.0001481 | 0.1001 | 0.0000148 | 27 | 0.85 | 0.0000126 |  |
| 0 | 1808 | 1808 | 1934 | 1327 | 0.0000885 | 0.1636 | 0.0000145 | 27 | 0.85 | 0.0000123 |  |
| 0 | 0 | 0 | -35 | -35 |  | 0 |  | 27 | 0.85 |  |  |

**Record of water head:**

| Times | Hour | Minute | Second | Start water head | Ended  water head | correction number | Total seconds | Corrects start water head | Corrects ended water head |
| --- | --- | --- | --- | --- | --- | --- | --- | --- | --- |
| 1 | 0 | 0 | 0 | 1590 |  | -35 | 0 | 1555 |  |
| 1 | 0 | 36 | 16 |  | 925 | -35 | 2176 |  | 890 |
| 2 | 0 | 0 | 0 | 1767 |  | -35 | 0 | 1732 |  |
| 2 | 0 | 38 | 13 |  | 1053 | -35 | 2293 |  | 1018 |
| 3 | 0 | 0 | 0 | 1839 |  | -35 | 0 | 1804 |  |
| 3 | 0 | 53 | 42 |  | 943 | -35 | 3222 |  | 908 |
| 4 | 0 | 0 | 0 | 1750 |  | -35 | 0 | 1715 |  |
| 4 | 0 | 34 | 24 |  | 1154 | -35 | 2064 |  | 1119 |
| 5 | 0 | 0 | 0 | 1968 |  | -35 | 0 | 1933 |  |
| 5 | 0 | 18 | 0 |  | 1570 | -35 | 1080 |  | 1535 |
| 6 | 0 | 0 | 0 | 1969 |  | -35 | 0 | 1934 |  |
| 6 | 0 | 30 | 8 |  | 1362 | -35 | 1808 |  | 1327 |
| 7 | 0 | 0 | 0 | 0 |  | -35 | 0 | -35 |  |
| 7 | 0 | 0 | 0 |  | 0 | -35 | 0 |  | -35 |

## (26) Water-absorbing content:0.00 g/m^2^; Adhesive content:0.00 g/m^2^; Weathered red-bed soil 0.00%:

| Start: t_1_ (min) | Finish: t_2_ | Duration: t | To start the water head: h1(cm) | End the water head:h_2_ (cm) | $2.3\frac{a}{A}\frac{L}{t}$ | $lg\frac{h_{1}}{h_{2}}$ | Permeability coefficient at water temperature T °C:k_T_ (cm/s) | Water temperature (℃) | Correction coefficient | Permeability coefficient: k_20_ (cm/s) | Average permeability coefficient:k_20_ (cm/s) |
| --- | --- | --- | --- | --- | --- | --- | --- | --- | --- | --- | --- |
| 1 | 2 | 3 | 4 | 5 | 6 | 7 | 8 | 9 | 10 | 11 | 12 |
|  |  | (2)-(1) |  |  | $2.3\frac{a}{A}\frac{L}{(3)}$ | $lg\frac{(4)}{(5)}$ | (6)×(7) |  |  | (8)×(10) | $\frac{\sum(11)}{n}$ |
| 0 | 119 | 119 | 1740 | 702 | 0.0013445 | 0.3945 | 0.0005304 | 26 | 0.87 | 0.0004614 | 0.00043 |
| 0 | 136 | 136 | 1800 | 665 | 0.0011765 | 0.4325 | 0.0005088 | 26 | 0.87 | 0.0004427 |  |
| 0 | 119 | 119 | 1770 | 765 | 0.0013445 | 0.3643 | 0.0004898 | 26 | 0.87 | 0.0004261 |  |
| 0 | 131 | 131 | 1699 | 672 | 0.0012214 | 0.4031 | 0.0004923 | 26 | 0.87 | 0.0004283 |  |
| 0 | 132 | 132 | 1691 | 678 | 0.0012121 | 0.3969 | 0.0004811 | 26 | 0.87 | 0.0004186 |  |
| 0 | 132 | 132 | 1805 | 737 | 0.0012121 | 0.389 | 0.0004715 | 26 | 0.87 | 0.0004102 |  |

**Record of water head:**

| Times | Hour | Minute | Second | Start water head | Ended  water head | correction number | Total seconds | Corrects start water head | Corrects ended water head |
| --- | --- | --- | --- | --- | --- | --- | --- | --- | --- |
| 1 | 0 | 0 | 0 | 1775 |  | -35 | 0 | 1740 |  |
| 1 | 0 | 1 | 59 |  | 737 | -35 | 119 |  | 702 |
| 2 | 0 | 0 | 0 | 1835 |  | -35 | 0 | 1800 |  |
| 2 | 0 | 2 | 16 |  | 700 | -35 | 136 |  | 665 |
| 3 | 0 | 0 | 0 | 1805 |  | -35 | 0 | 1770 |  |
| 3 | 0 | 1 | 59 |  | 800 | -35 | 119 |  | 765 |
| 4 | 0 | 0 | 0 | 1734 |  | -35 | 0 | 1699 |  |
| 4 | 0 | 2 | 11 |  | 707 | -35 | 131 |  | 672 |
| 5 | 0 | 0 | 0 | 1726 |  | -35 | 0 | 1691 |  |
| 5 | 0 | 2 | 12 |  | 713 | -35 | 132 |  | 678 |
| 6 | 0 | 0 | 0 | 1840 |  | -35 | 0 | 1805 |  |
| 6 | 0 | 2 | 12 |  | 772 | -35 | 132 |  | 737 |

## (27) Water-absorbing content:0.00 g/m^2^; Adhesive content:0.00 g/m^2^; Weathered red-bed soil 1.00%:

| Start: t_1_ (min) | Finish: t_2_ | Duration: t | To start the water head: h1(cm) | End the water head:h_2_ (cm) | $2.3\frac{a}{A}\frac{L}{t}$ | $lg\frac{h_{1}}{h_{2}}$ | Permeability coefficient at water temperature T °C:k_T_ (cm/s) | Water temperature (℃) | Correction coefficient | Permeability coefficient: k_20_ (cm/s) | Average permeability coefficient:k_20_ (cm/s) |
| --- | --- | --- | --- | --- | --- | --- | --- | --- | --- | --- | --- |
| 1 | 2 | 3 | 4 | 5 | 6 | 7 | 8 | 9 | 10 | 11 | 12 |
|  |  | (2)-(1) |  |  | $2.3\frac{a}{A}\frac{L}{(3)}$ | $lg\frac{(4)}{(5)}$ | (6)×(7) |  |  | (8)×(10) | $\frac{\sum(11)}{n}$ |
| 0 | 4213 | 4213 | 1955 | 777 | 0.000038 | 0.4014 | 0.0000153 | 27 | 0.85 | 0.000013 | 1.24E-05 |
| 0 | 2517 | 2517 | 1846 | 1081 | 0.0000636 | 0.2324 | 0.0000148 | 27 | 0.85 | 0.0000126 |  |
| 0 | 2192 | 2192 | 1702 | 1080 | 0.000073 | 0.1975 | 0.0000144 | 27 | 0.85 | 0.0000122 |  |
| 0 | 3867 | 3867 | 1880 | 866 | 0.0000414 | 0.3365 | 0.0000139 | 27 | 0.85 | 0.0000118 |  |
| 0 | 1166 | 1166 | 1909 | 1151 | 0.0001372 | 0.2197 | 0.0000301 | 27 |  |  |  |
| 0 | 2605 | 2605 | 1909 | 624 | 0.0000614 | 0.4856 | 0.0000298 | 27 |  |  |  |
| 0 | 1041 | 1041 | 1675 | 345 | 0.0001537 | 0.6862 | 0.0001055 | 17 |  |  |  |

**Record of water head:**

| Times | Hour | Minute | Second | Start water head | Ended  water head | correction number | Total seconds | Corrects start water head | Corrects ended water head |
| --- | --- | --- | --- | --- | --- | --- | --- | --- | --- |
| 1 | 0 | 0 | 0 | 1995 |  | -40 | 0 | 1955 |  |
| 1 | 1 | 10 | 13 |  | 817 | -40 | 4213 |  | 777 |
| 2 | 0 | 0 | 0 | 1886 |  | -40 | 0 | 1846 |  |
| 2 | 0 | 41 | 57 |  | 1121 | -40 | 2517 |  | 1081 |
| 3 | 0 | 0 | 0 | 1742 |  | -40 | 0 | 1702 |  |
| 3 | 0 | 36 | 32 |  | 1120 | -40 | 2192 |  | 1080 |
| 4 | 0 | 0 | 0 | 1920 |  | -40 | 0 | 1880 |  |
| 4 | 1 | 4 | 27 |  | 906 | -40 | 3867 |  | 866 |
| 5 | 0 | 0 | 0 | 1939 |  | -30 | 0 | 1909 |  |
| 5 | 0 | 19 | 26 |  | 1181 | -30 | 1166 |  | 1151 |
| 6 | 0 | 0 | 0 | 1939 |  | -30 | 0 | 1909 |  |
| 6 | 0 | 43 | 25 |  | 654 | -30 | 2605 |  | 624 |
| 7 | 0 | 0 | 0 | 1705 |  | -30 | 0 | 1675 |  |
| 7 | 0 | 17 | 21 |  | 375 | -30 | 1041 |  | 345 |

## (28) Water-absorbing content:0.00 g/m^2^; Adhesive content:0.00 g/m^2^; Weathered red-bed soil 2.50%:

| Start: t_1_ (min) | Finish: t_2_ | Duration: t | To start the water head: h1(cm) | End the water head:h_2_ (cm) | $2.3\frac{a}{A}\frac{L}{t}$ | $lg\frac{h_{1}}{h_{2}}$ | Permeability coefficient at water temperature T °C:k_T_ (cm/s) | Water temperature (℃) | Correction coefficient | Permeability coefficient: k_20_ (cm/s) | Average permeability coefficient:k_20_ (cm/s) |
| --- | --- | --- | --- | --- | --- | --- | --- | --- | --- | --- | --- |
| 1 | 2 | 3 | 4 | 5 | 6 | 7 | 8 | 9 | 10 | 11 | 12 |
|  |  | (2)-(1) |  |  | $2.3\frac{a}{A}\frac{L}{(3)}$ | $lg\frac{(4)}{(5)}$ | (6)×(7) |  |  | (8)×(10) | $\frac{\sum(11)}{n}$ |
| 0 | 1459 | 1459 | 1642 | 850 | 0.0001097 | 0.2856 | 0.0000313 | 29 | 0.815 | 0.0000255 | 0.0000267 |
| 0 | 886 | 886 | 1930 | 1269 | 0.0001806 | 0.1821 | 0.0000329 | 29 | 0.815 | 0.0000268 |  |
| 0 | 966 | 966 | 1701 | 1088 | 0.0001656 | 0.1941 | 0.0000321 | 27 | 0.85 | 0.0000273 |  |
| 0 | 2421 | 2421 | 1910 | 560 | 0.0000661 | 0.5328 | 0.0000352 | 27 | 0.85 | 0.0000299 |  |
| 0 | 1166 | 1166 | 1909 | 1151 | 0.0001372 | 0.2197 | 0.0000301 | 27 | 0.85 | 0.0000256 |  |
| 0 | 2605 | 2605 | 1909 | 624 | 0.0000614 | 0.4856 | 0.0000298 | 27 | 0.85 | 0.0000253 |  |
| 0 | 1041 | 1041 | 1675 | 345 | 0.0001537 | 0.6862 | 0.0001055 | 17 | 1.077 | 0.0001136 |  |

**Record of water head:**

| Times | Hour | Minute | Second | Start water head | Ended  water head | correction number | Total seconds | Corrects start water head | Corrects ended water head |
| --- | --- | --- | --- | --- | --- | --- | --- | --- | --- |
| 1 | 0 | 0 | 0 | 1672 |  | -30 | 0 | 1642 |  |
| 1 | 0 | 24 | 19 |  | 880 | -30 | 1459 |  | 850 |
| 2 | 0 | 0 | 0 | 1960 |  | -30 | 0 | 1930 |  |
| 2 | 0 | 14 | 46 |  | 1299 | -30 | 886 |  | 1269 |
| 3 | 0 | 0 | 0 | 1731 |  | -30 | 0 | 1701 |  |
| 3 | 0 | 16 | 6 |  | 1118 | -30 | 966 |  | 1088 |
| 4 | 0 | 0 | 0 | 1940 |  | -30 | 0 | 1910 |  |
| 4 | 0 | 40 | 21 |  | 590 | -30 | 2421 |  | 560 |
| 5 | 0 | 0 | 0 | 1939 |  | -30 | 0 | 1909 |  |
| 5 | 0 | 19 | 26 |  | 1181 | -30 | 1166 |  | 1151 |
| 6 | 0 | 0 | 0 | 1939 |  | -30 | 0 | 1909 |  |
| 6 | 0 | 43 | 25 |  | 654 | -30 | 2605 |  | 624 |
| 7 | 0 | 0 | 0 | 1705 |  | -30 | 0 | 1675 |  |
| 7 | 0 | 17 | 21 |  | 375 | -30 | 1041 |  | 345 |

## (29) Water-absorbing content: 0.00 g/m^2^; Adhesive content:0.00 g/m^2^; Weathered red-bed soil 0.00%:

| Start: t_1_ (min) | Finish: t_2_ | Duration: t | To start the water head: h1(cm) | End the water head:h_2_ (cm) | $2.3\frac{a}{A}\frac{L}{t}$ | $lg\frac{h_{1}}{h_{2}}$ | Permeability coefficient at water temperature T °C:k_T_ (cm/s) | Water temperature (℃) | Correction coefficient | Permeability coefficient: k_20_ (cm/s) | Average permeability coefficient:k_20_ (cm/s) |
| --- | --- | --- | --- | --- | --- | --- | --- | --- | --- | --- | --- |
| 1 | 2 | 3 | 4 | 5 | 6 | 7 | 8 | 9 | 10 | 11 | 12 |
|  |  | (2)-(1) |  |  | $2.3\frac{a}{A}\frac{L}{(3)}$ | $lg\frac{(4)}{(5)}$ | (6)×(7) |  |  | (8)×(10) | $\frac{\sum(11)}{n}$ |
|  |  | (2)-(1) |  |  |  |  | (6)×(7) |  |  | (8)×(10) | 0.0000548 |
| 0 | 930 | 930 | 1810 | 780 | 0.000172 | 0.3655 | 0.0000629 | 26 | 0.87 | 0.0000547 |  |
| 0 | 959 | 959 | 1760 | 694 | 0.0001668 | 0.4042 | 0.0000674 | 26 | 0.87 | 0.0000586 |  |
| 0 | 1037 | 1037 | 1861 | 713 | 0.0001543 | 0.4167 | 0.0000643 | 26 | 0.87 | 0.0000559 |  |
| 0 | 1006 | 1006 | 1730 | 700 | 0.0001590 | 0.3927 | 0.0000624 | 26 | 0.87 | 0.0000543 |  |
| 0 | 873 | 873 | 1760 | 818 | 0.0001833 | 0.3328 | 0.000061 | 26 | 0.87 | 0.0000531 |  |
| 0 | 906 | 906 | 1750 | 802 | 0.0001766 | 0.3389 | 0.0000598 | 26 | 0.87 | 0.000052 |  |

**Record of water head:**

| Times | Hour | Minute | Second | Start water head | Ended  water head | correction number | Total seconds | Corrects start water head | Corrects ended water head |
| --- | --- | --- | --- | --- | --- | --- | --- | --- | --- |
| 1 | 0 | 0 | 0 | 1840 |  | -30 | 0 | 1810 |  |
| 1 | 0 | 15 | 30 |  | 810 | -30 | 930 |  | 780 |
| 2 | 0 | 0 | 0 | 1790 |  | -30 | 0 | 1760 |  |
| 2 | 0 | 15 | 59 |  | 724 | -30 | 959 |  | 694 |
| 3 | 0 | 0 | 0 | 1891 |  | -30 | 0 | 1861 |  |
| 3 | 0 | 17 | 17 |  | 743 | -30 | 1037 |  | 713 |
| 4 | 0 | 0 | 0 | 1760 |  | -30 | 0 | 1730 |  |
| 4 | 0 | 16 | 46 |  | 730 | -30 | 1006 |  | 700 |
| 5 | 0 | 0 | 0 | 1790 |  | -30 | 0 | 1760 |  |
| 5 | 0 | 14 | 33 |  | 848 | -30 | 873 |  | 818 |
| 6 | 0 | 0 | 0 | 1780 |  | -30 | 0 | 1750 |  |
| 6 | 0 | 15 | 6 |  | 832 | -30 | 906 |  | 802 |
| 7 | 0 | 0 | 0 | 1705 |  | 430 | 0 | 2135 |  |
| 7 | 1 | 17 | 21 |  | 375 | 430 | 4641 |  | 805 |

## (10) Water-absorbing content:30.00 g/m^2^; Adhesive content:5.00 g/m^2^; Weathered red-bed soil 5.00%:

| Start: t_1_ (min) | Finish: t_2_ | Duration: t | To start the water head: h1(cm) | End the water head:h_2_ (cm) | $2.3\frac{a}{A}\frac{L}{t}$ | $lg\frac{h_{1}}{h_{2}}$ | Permeability coefficient at water temperature T °C:k_T_ (cm/s) | Water temperature (℃) | Correction coefficient | Permeability coefficient: k_20_ (cm/s) | Average permeability coefficient:k_20_ (cm/s) |
| --- | --- | --- | --- | --- | --- | --- | --- | --- | --- | --- | --- |
| 1 | 2 | 3 | 4 | 5 | 6 | 7 | 8 | 9 | 10 | 11 | 12 |
|  |  | (2)-(1) |  |  | $2.3\frac{a}{A}\frac{L}{(3)}$ | $lg\frac{(4)}{(5)}$ | (6)×(7) |  |  | (8)×(10) | $\frac{\sum(11)}{n}$ |
| 0 | 2982 | 2982 | 1733 | 801 | 0.0000537 | 0.3345 | 0.000018 | 26 | 0.87 | 0.0000157 | 0.0000152 |
| 0 | 1965 | 1965 | 1724 | 1051 | 0.0000814 | 0.2149 | 0.0000175 | 26 | 0.87 | 0.0000152 |  |
| 0 | 856 | 856 | 1786 | 1441 | 0.0001869 | 0.0932 | 0.0000174 | 26 | 0.87 | 0.0000151 |  |
| 0 | 1607 | 1607 | 1960 | 1320 | 0.0000996 | 0.1703 | 0.000017 | 26 | 0.87 | 0.0000148 |  |
| 0 | 1672 | 1672 | 1905 | 272 | 0.0000957 | 0.8453 | 0.0000809 | 26 | 0.87 | 0.0000704 |  |
| 0 | 2110 | 2110 | 1600 | 750 | 0.0000758 | 0.3291 | 0.0000249 | 26 | 0.87 | 0.0000217 |  |
| 0 | 4641 | 4641 | 2135 | 805 | 0.0000345 | 0.4236 | 0.0000146 | 17 |  |  |  |

**Record of water head:**

| Times | Hour | Minute | Second | Start water head | Ended  water head | correction number | Total seconds | Corrects start water head | Corrects ended water head |
| --- | --- | --- | --- | --- | --- | --- | --- | --- | --- |
| 1 | 0 | 0 | 0 | 1773 |  | -40 | 0 | 1733 |  |
| 1 | 0 | 49 | 42 |  | 841 | -40 | 2982 |  | 801 |
| 2 | 0 | 0 | 0 | 1764 |  | -40 | 0 | 1724 |  |
| 2 | 0 | 32 | 45 |  | 1091 | -40 | 1965 |  | 1051 |
| 3 | 0 | 0 | 0 | 1826 |  | -40 | 0 | 1786 |  |
| 3 | 0 | 14 | 16 |  | 1481 | -40 | 856 |  | 1441 |
| 4 | 0 | 0 | 0 | 2000 |  | -40 | 0 | 1960 |  |
| 4 | 0 | 26 | 47 |  | 1360 | -40 | 1607 |  | 1320 |
| 5 | 0 | 0 | 0 | 1945 |  | -40 | 0 | 1905 |  |
| 5 | 0 | 27 | 52 |  | 312 | -40 | 1672 |  | 272 |
| 6 | 0 | 0 | 0 | 1640 |  | -40 | 0 | 1600 |  |
| 6 | 0 | 35 | 10 |  | 790 | -40 | 2110 |  | 750 |
| 7 | 0 | 0 | 0 | 1705 |  | 430 | 0 | 2135 |  |
| 7 | 1 | 17 | 21 |  | 375 | 430 | 4641 |  | 805 |

# Fig 15. (a) Upper and lower limitations applied (cm/s)

| **materials** | **0.00/0.00 (g/m^2^)** | **15.00/2.50 (g/m^2^)** | **15.00/5.00 (g/m^2^)** | **30.00/2.50 (g/m^2^)** | **30.00/5.00 (g/m^2^)** | **60.00/10.00 (g/m^2^)** |
| --- | --- | --- | --- | --- | --- | --- |
| **0.00 (%)** | 0.43 | 0.46 | 0.56 | 0.43 | 1.21 | 2.16 |
| **1.00 (%)** | 0.12 | 0.67 | 0.45 | 0.23 | 1.20 | 1.83 |
| **2.50 (%)** | 0.27 | 0.57 | 0.61 | 0.43 | 0.38 | 1.36 |
| **5.00 (%)** | 0.27 | 0.34 | 0.41 | 0.43 | 0.58 | 0.66 |
| **10.00 (%)** | 0.15 | 0.33 | 0.13 | 0.52 | 0.34 | 0.59 |

# Fig 15. (b) Upper and lower limitations not applied.

| **materials** | **0.00/0.00 (g/m^2^)** | **15.00/2.50 (g/m^2^)** | **15.00/5.00 (g/m^2^)** | **30.00/2.50 (g/m^2^)** | **30.00/5.00 (g/m^2^)** | **60.00/10.00 (g/m^2^)** |
| --- | --- | --- | --- | --- | --- | --- |
| **0.00 (%)** | 0.31 | 0.28 | 0.38 | 0.40 | 0.66 | 0.78 |
| **1.00 (%)** | 0.43 | 0.34 | 0.41 | 0.47 | 0.78 | 1.18 |
| **2.50 (%)** | 0.43 | 0.43 | 0.50 | 0.71 | 0.63 | 1.14 |
| **5.00 (%)** | 0.56 | 0.60 | 0.55 | 0.34 | 0.87 | 1.19 |
| **10.00 (%)** | 0.67 | 0.62 | 0.87 | 1.01 | 0.92 | 1.72 |

# Germination rate calculation

| **materials** | **0.00/0.00 (g/m^2^)** | | **15.00/2.50 (g/m^2^)** | | **15.00/5.00 (g/m^2^)** | | **30.00/2.50 (g/m^2^)** | | **30.00/5.00 (g/m^2^)** | | **60.00/10.00 (g/m^2^)** | |
| --- | --- | --- | --- | --- | --- | --- | --- | --- | --- | --- | --- | --- |
|  | number | rate | number | rate | number | rate | number | rate | number | rate | number | rate |
| **0.00 (%)** | 8 | 40 | 16 | 80 | 8 | 40 | 15 | 75 | 13 | 65 | 7 | 35 |
| **1.00 (%)** | 15 | 75 | 15 | 75 | 16 | 80 | 15 | 75 | 10 | 50 | 13 | 65 |
| **2.50 (%)** | 13 | 65 | 9 | 45 | 15 | 75 | 16 | 80 | 12 | 60 | 17 | 85 |
| **5.00 (%)** | 18 | 90 | 16 | 80 | 14 | 70 | 7 | 35 | 11 | 55 | 14 | 70 |
| **10.00 (%)** | 19 | 95 | 15 | 75 | 16 | 80 | 17 | 85 | 18 | 90 | 13 | 65 |

# 25th day average plant height (mm)

| **Group number** | **Plant height on the 25th day (mm)** | | | |
| --- | --- | --- | --- | --- |
|  | **Measurement 1** | **Measurement 2** | **Measurement 3** | **Mean value** |
| 1 | 105 | 109 | 101 | 105.00 |
| 2 | 123 | 126 | 126 | 125.00 |
| 3 | 127 | 127 | 127 | 127.00 |
| 4 | 123 | 125 | 124 | 124.00 |
| 5 | 135 | 139 | 131 | 135.00 |
| 6 | 110 | 107 | 117 | 112.00 |
| 7 | 115 | 120 | 121 | 118.00 |
| 8 | 127 | 125 | 126 | 126.00 |
| 9 | 130 | 124 | 124 | 126.00 |
| 10 | 100 | 98 | 105 | 101.00 |

# Fig 17. Temporal change in plant growth in ester materials coupled with weathered red-bed soil

| **Group number** | **Germination rate (%)** | **Plant height on the 25th day (mm)** |
| --- | --- | --- |
| 1 | 50.00 | 105.00 |
| 2 | 80.00 | 125.00 |
| 3 | 75.00 | 127.00 |
| 4 | 75.00 | 124.00 |
| 5 | 90.00 | 135.00 |
| 6 | 65.00 | 112.00 |
| 7 | 65.00 | 118.00 |
| 8 | 70.00 | 126.00 |
| 9 | 80.00 | 126.00 |
| 10 | 45.00 | 101.00 |
| 11 | 35.00 | 98.00 |
