# Supplementary material for: The performance of partially substituted composite ester materials with weathered red-bed soil in ecological restoration
Source: PLoS One. 2024 Apr 3;19(4):e0299323. doi: 10.1371/journal.pone.0299323 (PMC10990215; doi:10.1371/journal.pone.0299323)
Supplement: Attachment — Submitted filename: Response to Reviewers.docx [file pone.0299323.s002.docx]

**Journal Requirements:**

**Point 1:** When submitting your revision, we need you to address these additional requirements. Please ensure that your manuscript meets PLOS ONE's style requirements, including those for file naming.

**Response 1：** Thank you for reminding me. We have modified the format of this article according to the template you provided.

**Point 2:** In your Methods section, please provide additional information regarding the permits you obtained for the work. Please ensure you have included the full name of the authority that approved the field site access and, if no permits were required, a brief statement explaining why.

**Response 2:** Thank you very much for your review of this article and your valuable comments. This research work was tolerated and supported by CCCC FOURTH HARBOR ENGINEERING CO., LTD. We have added the project number supported by CCCC FOURTH HARBOR ENGINEERING CO., LTD. to the project grant: 20187614071020007.

**Point 3:** Thank you for stating in your Funding Statement: The research is supported by the National Natural Science Foundation of China (NSFC) (Grant Numbers: 42293354, 42293351, 42293355, 42277131, 41977230).

Please provide an amended statement that declares all the funding or sources of support (whether external or internal to your organization) received during this study, as detailed online in our guide for authors at http://journals.plos.org/plosone/s/submit-now. Please also include the statement “There was no additional external funding received for this study.” in your updated Funding Statement.

Please include your amended Funding Statement within your cover letter. We will change the online submission form on your behalf.

**Response 3:** We have stated in the cover letter as follows“This research is supported by the National Natural Science Foundation of China (Grant Numbers: 42293354, 42293351, 42293355, 42277131, 41977230). These fundings are all awarded by the corresponding author Cuiying Zhou. We declare

that the funder has no known competing financial interests or personal relationships that could have appeared to influence the work reported in this paper. The funders had no role in study design, data collection and analysis, decision to publish, or preparation of the manuscript.”

**Point 4:** Thank you for stating the following in the Acknowledgments Section of your manuscript:

The research is supported by the National Natural Science Foundation of China (NSFC) (Grant Numbers: 42293354, 42293351, 42293355, 42277131, 41977230).

We note that you have provided funding information that is not currently declared in your Funding Statement. However, funding information should not appear in the Acknowledgments section or other areas of your manuscript. We will only publish funding information present in the Funding Statement section of the online submission form.

Please remove any funding-related text from the manuscript and let us know how you would like to update your Funding Statement. Currently, your Funding Statement reads as follows:

The research is supported by the National Natural Science Foundation of China (NSFC) (Grant Numbers: 42293354, 42293351, 42293355, 42277131, 41977230).

Please include your amended statements within your cover letter; we will change the online submission form on your behalf.

**Response 4:** Thanks for reminding me. The funding information has been removed from the acknowledgements section of the manuscript and included in the cover letter.

**Point 5:** In your Data Availability statement, you have not specified where the minimal data set underlying the results described in your manuscript can be found. PLOS defines a study's minimal data set as the underlying data used to reach the conclusions drawn in the manuscript and any additional data required to replicate the reported study findings in their entirety. All PLOS journals require that the minimal data set be made fully available. For more information about our data policy, please see <http://journals.plos.org/plosone/s/data-availability>.

Upon re-submitting your revised manuscript, please upload your study’s minimal underlying data set as either Supporting Information files or to a stable, public repository and include the relevant URLs, DOIs, or accession numbers within your revised cover letter. For a list of acceptable repositories, please see http://journals.plos.org/plosone/s/data-availability#loc-recommended-repositories. Any potentially identifying patient information must be fully anonymized.

**Response 5:** Thank you for reminding me. Relevant data supporting the results of this study have been uploaded to a file named " ecological_restoration ".

**Comments and Suggestions for Authors：**

This paper introduces an interesting approach of incorporating weathered red-bed soil as a partial alternative to composite ester materials for ecological restoration. The results show that the developed method has the potential to reduce the cost of ecological restoration by using specific ecological restoration indicators. This is an interesting study and paper can be accepted for publication after some revisions.

**Response:** Thank you very much for your review of this article and your valuable comments. The author has modified the full text one by one according to your Suggestions. The specific modified content and the reply to the question are shown below.

**Point 1:** The Abstract should be fortified by including specific quantitative results to provide a clearer snapshot of the findings.

**Response 1:** Thank you very much for your review of this article and your valuable comments. We have added the quantitative results of the trial in the abstract section. You can see the supplementary content in **L16-19**.

**Point 2:** Within the Introduction, refrain from clustering multiple references at the end of a single sentence. Each cited study offers distinct insights; thus, summarizing the pivotal findings of each will guide readers through the evolution and direction of the research field. In the concluding portion of the Introduction, the authors must distinctly highlight the unique contributions and innovations of their research.

**Response 2:** Thank you very much for your review of this article and your valuable comments. We summarize the right and wrong of the works cited. The contribution of these works to this study is also analyzed and what corrections need to be made on this basis. You can see the supplementary content in **L41-74**.

**Point 3:** While the authors embarked on a dual test of the composite properties and ecological impact of the weathered red-bed soil and ester materials, the rationale for this approach wasn't provided. Clarifying the purpose and significance of this test will ensure readers grasp its relevance.

**Response 3:** Thank you very much for your review of this article and your valuable comments. We explain the purpose and significance of this test in the article. You can see the supplementary content in **L146-149** and **L243-245**. And it also provides the rationale for this approach. You can see the supplementary content in **L155** and **L179**.

**Point 4:** In the section discussing research content and methods, omit sentences that only serve as basic introductions.

**Response 4:** Thank you very much for your review of this article and your valuable comments. We have checked the article text and omit sentences that only serve as basic introductions.

**Point 5:** In the research content and methods section, the determination of the optimal ratio solely based on plant growth indicators from the ester material compounding experiment is limiting. To provide a more comprehensive insight, it would be prudent to consider all indicators listed in Table 1.

**Response 5:** Thank you very much for your review of this article and your valuable comments. We have considered all the indicators in Table 1 in the ester material composite experiment You can see the supplementary content in **L146-149** and **Fig 4b**.

**Point 6:** Ensure that figure captions align with their respective mentions in the manuscript. Specifically, there's a discrepancy between Figure 9 and its corresponding reference in the text. Additionally, confirm the correctness of units and the layout of all tables and figures.

**Response 6:** Thank you very much for your review of this article and your valuable comments.

We have mapped the figure captions to the respective mentions in the manuscript and confirmed the correctness of the units and the layout of all tables and figures. You can see the supplement content in the full text.

**Point 7:** Throughout the paper, it's observed that there's inconsistency in the presentation of significant figures and decimals (e.g., discrepancies between 1.52 and 1.21 in Table 1; 29.98 and 28.6 in Figure 10). Standardizing these figures will enhance the manuscript's precision.

**Response 7:** Thank you very much for your review of this article and your valuable comments. We apologize for not noticing this. We have standardized the significant figures throughout the paper. You can see the revisions in the text.

**Point 8:** Figure 6, which delineates the cohesion variation in different ester materials with the inclusion of weathered red-bed soil, should be represented more accurately. Instead of mere curve connections, a fitting method that more closely mirrors the actual change would be more appropriate.

**Response 8:** Thank you very much for your review of this article and your valuable comments. We have used Modified Bezier curve fitting in Figure 6, and the fitting equation can be seen in Figure 6.

**Point 9:** In the Discussion, there's a palpable gap between the proposed mechanism and the derived experimental data. The proposed explanations lean heavily on inferred logic. To solidify the credibility of the proposed mechanism, drawing more overt correlations between the experimental data and the given interpretations is essential.

**Response 9:** Thank you very much for your review of this article and your valuable comments. We have linked the correlation between the proposed mechanism and the derived experimental data. You can see the supplementary content in **L381-420**.

**Point 10:** In the Conclusion, initiate with a general statement before diving into point-wise findings.

**Response 10:** Thank you very much for your review of this article and your valuable comments. We have started with a general statement, followed by a point-by-point conclusion. You can see the supplementary content in **L462-487**.
