# Supplementary material for: The performance of partially substituted composite ester materials with weathered red-bed soil in ecological restoration
Source: PLoS One. 2024 Apr 3;19(4):e0299323. doi: 10.1371/journal.pone.0299323 (PMC10990215; doi:10.1371/journal.pone.0299323)
Supplement: Attachment — Submitted filename: Response to Reviewers.docx [file pone.0299323.s004.docx]

**Comments and Suggestions for Authors：**

The manuscript deals with the performance of partially substituted composite ester materials with weathered red bed soil in ecological restoration. Referring to the revised manuscript and comments provided by the authors, the following changes have been recommended. The article can be considered for publication after making the following changes.

**Response:** Thank you very much for your review of this article and your valuable comments. The author has modified the full text one by one according to your Suggestions. The specific modified content and the reply to the question are shown below.

**Point 1:** The entire manuscript is recommended for a careful and thorough check by a certified proofreading agency to ensure that the revised article is free from grammatical mistakes, spelling mistakes, punctuation errors, etc. The authors have to place the superscripts and subscripts throughout the content correctly.

**Response 1:** We have polished the entire manuscript in the editing agency and corrected grammar, spelling, punctuation, and superscript and subscript issues throughout the manuscript. You can see the corrections in the manuscript.

**Point 2:** The sentence in lines 17-19 does not provide clarity and the authors are requested to modify it as “The experimental findings indicated that the soil modified using ester materials exhibited an improvement in strength, water retention, and aeration due to change in the soil structure”.

**Response 2:** We have revised the sentence from lines 17 to 19 to modify: "Experimental results show that due to changes in soil structure, soil modified with ester materials exhibits improvements in strength, water retention, and aeration.". You can see the supplementary content in **L17-19**.

**Point 3:** Introduction: The authors haven’t mentioned ester materials previously used for soil improvement. Rather, only the references have been cited.

**Response 3:** We have summarized the ester materials previously used for soil improvement and cited corresponding references in the introduction. You can see the supplementary content in **L32-33**.

**Point 4:** In Table 3, the authors can cite the relevant references against each point rather than citing them against the title.

**Response 4:** We have cited relevant references for each point in Table 3. You can see the supplementary content in **Table 3.**

**Point 5:** Figures 2, 9, and 12 need to be replaced with better-quality images.

**Response 5:** We have replaced Figures 2, 9, and 12 with better quality images. You can see the supplementary content in **Figures 2, 9, and 12.**
